# Supplementary material for: Do childhood socioeconomic circumstances moderate the association between childhood cognitive ability and all-cause mortality across the life course? Prospective observational study of the 36-day sample of the Scottish Mental Survey 1947
Source: BMJ Open. 2020 Dec 2;10(12):e037847. doi: 10.1136/bmjopen-2020-037847 (PMC7712420; doi:10.1136/bmjopen-2020-037847)
Supplement: Supplementary data [file bmjopen-2020-037847supp001.pdf]

Supplementary Material

Bias analyses

Although the analyses presented in the main text incorporates sex and age as covariates, there is the potential for unmeasured factors to confound the observed associations with mortality risk. To account for this, we calculated E-values to estimate the minimum strength of association that an unmeasured confounder would need to have with each predictor (age-11 IQ or father's social class) and mortality risk, on the hazard ratio (HR) scale, to fully explain the observed associations. Large E-values indicate that strong confounding would be required to fully account for a given association. Notably, it is recommended that E-values are reported both for a given point estimate HR and for its 95% CI boundary closest to the null (1 on the HR scale)(1).

Based on the univariate association between age-11 IQ and mortality risk (Main Text, Table 2), we estimated an E-value of 1.58 (E-value for upper limit of 95% CI = 1.51). Based on the sex- and age-adjusted association between age-11 IQ and mortality risk (Main Text, Table 2), we estimated an E-value of 1.58 (E-value for upper limit of 95% CI = 1.48). Finally, based on the point estimate of the mutually-adjusted (sex, age and father's social class) association between age-11 IQ and mortality risk (Main Text, Table 2), we estimated an E-value of 1.56 (E-value for upper limit of 95% CI = 1.46).

Based on the univariate association between father's social class and mortality risk (Main Text, Table 2), we estimated an E-value of 1.39 (E-value for upper limit of 95% CI = 1.28). Based on the sex- and age-adjusted association between father's social class and mortality risk (Main Text, Table 2), we estimated an E-value of 1.39 (E-value for upper limit of 95% CI = 1.28). Finally, based on the point estimate of the mutually-adjusted (sex, age and father's social class) association between father's social class and mortality risk (Main Text, Table 2), we estimated an E-value of 1.28 (E-value for upper limit of 95% CI = 1.17).

In the context of the associations observed in the present study, this analysis suggests that a potential confounder would need to have a relatively strong association with the predictors and mortality risk to fully explain their association, particularly the association between age-11 IQ and mortality risk.

Unadjusted interaction analyses

The interaction analyses reported in the main text are adjusted for sex and age in days at Scottish Mental Survey 1947. In the analyses presented below (Table A1) we re-estimate the mutually-adjusted model (including IQ score and father's social class) and the interaction model (including the IQ X social class interaction) without adjusting for these potential confounders, and with adjusting for only one confounder (sex or age).

Adding the IQ-social class interaction term did not significantly improve model fit versus the corresponding main effects model (Unadjusted: AIC = 35357.72, $X^2\Delta$ = 0.95, p = 0.33; Sex-adjusted: AIC = 35270.27, $X^2\Delta$ = 1.36, p = 0.24; Age-adjusted: AIC = 35356.28, $X^2\Delta$ = 0.96, p = 0.33). Furthermore, the interaction did not significantly predict all-cause mortality in any of the models (see Table A1).

Table A1. Results from weighted Cox regression models predicting all-cause mortality risk. Regression coefficients and hazard ratios (HR) for IQ score and Father's social class in main effects only model and the interaction model without further adjustment for covariates, adjusted for sex only, and adjusted for age at Scottish Mental Survey 1947 only.

	Unadjusted[1,2]				Sex-adjusted[3,4]				Age-adjusted[5,6]			
**Predictor**	**B**	**SE**	**HR [95% CI]**	**p**	**B**	**SE**	**HR [95% CI]**	**p**	**B**	**SE**	**HR [95% CI]**	**p**
Main effects only model												
IQ score (1 SD)	-0.20	0.02	0.82 [0.79-0.86]	<0.001	-0.19	0.02	0.83 [0.79-0.86]	<0.001	-0.21	0.02	0.81 [0.78-0.85]	<0.001
Father's social class (1 class)	-0.08	0.02	0.93 [0.89-0.97]	<0.001	-0.08	0.02	0.92 [0.88-0.97]	<0.001	-0.07	0.02	0.93 [0.89-0.97]	<0.01
Interaction model												
IQ score X Father's social class	0.02	0.02	1.02 [0.98-1.07]	0.33	0.03	0.02	1.03 [0.98-1.07]	0.24	0.02	0.02	1.02 [0.98-1.07]	0.33

[1]Unadjusted main effects only model R^2 = 0.02; [2]Unadjusted interaction model R^2 = 0.02; [3]Sex-adjusted main effects only mode R^2 = 0.04; [4]Sex-adjusted interaction model R^2 = 0.04; [5]Age-adjusted main effects only model R^2 = 0.02, [6]Age-adjusted interaction model R^2 = 0.02.

3-way Interaction

At the request of a reviewer, we additionally performed an exploratory analysis of a three-way interaction between IQ z-scores, father's occupational social class (reversed, continuous) and sex. In particular, the aim was to test whether the moderating role of childhood socioeconomic circumstances itself depended on sex.

This was done by constructing a Cox regression model that included main effects of IQ z-scores, father's social class (reversed, continuous), sex and age in days at the Scottish Mental Survey 1947, two-way interactions between IQ z-scores X father's social class, IQ z-scores X sex and father's social class X sex, as well as the three-way interaction between IQ z-scores X father's social class X sex.

Including these additional interaction effects resulted in multicollinearity issues (all interaction VIFs > 8.50). Furthermore, it did not significantly improve model fit versus the IQ z-score X father's social class model included in the main text (AIC = 35275.82; $X^2\Delta = 0.26$, p = 0.97; Interaction $R^2 = 0.04$). None of the included interactions significantly predicted all-cause mortality risk (Table A2).

Table A2. Results from weighted Cox regression model, adjusted for age in days at Scottish Mental Survey 1947, predicting all-cause mortality risk. Regression coefficients and hazard ratios (HR) for IQ score, Father's social class, Sex, IQ X Father's social class, IQ X Sex, Father's social class X Sex, IQ X Father's social class X Sex.

**Predictor**	**B**	**SE**	**HR [95% CI]**	**p**
IQ score (1 SD)	-0.26	0.07	0.77 [0.67-0.89]	<0.001
Father's social class (1 class)	-0.07	0.03	0.94 [0.88-1.00]	0.04
Sex (Female)	-0.36	0.13	0.70 [0.54-0.90]	<0.01
IQ X Father's social class	0.02	0.03	1.03 [0.97-1.08]	0.38
IQ X Sex	0.01	0.12	1.01 [0.80-1.27]	0.94
Father's social class X Sex	-0.02	0.05	0.98 [0.89-1.08]	0.70
IQ X Father's social class X Sex	<0.01	0.04	1.00 [0.92-1.10]	0.94

Additive hazards model

At the request of a reviewer, we performed Additive Hazards regression using Aalen's additive regression models equivalent to the Cox Proportional Hazards models used in the main text. Additive models have the advantage of allowing the effects of covariates – including interaction effects – to vary over time (2). For example, advantage in childhood cognitive ability may benefit survival in particular socioeconomic groups but only in specific periods of life. Additive models help to provide information about the effect in the context of the underlying hazard – a small hazard ratio for the interaction may still be important if the

underlying hazard is large (2). We estimated the number of additional deaths per 10,000 person years predicted by each covariate of interest – IQ z-scores, father's occupational social class and the IQ-social class interaction. As in the main text, univariate, sex and age-adjusted, mutually-adjusted, and interaction models were constructed.

As with mortality risk in the main text, a 1SD advantage in IQ and a 1 class advantage in father's social class were both significantly associated with a small reduction in the number of deaths, even when adjusted for sex and age at time of the Scottish Mental Survey 1947 (Table A3). These associations remained significant when mutually-adjusting, supporting the suggestion that childhood cognitive ability and childhood socioeconomic circumstances independently predict mortality risk.

We then tested the moderating effect of father's social class. The IQ-social class interaction predicted a small increase of 0.13 deaths per 10,000 person years, though this was only marginally-significant and 95% CIs included 0 (95% CI [0.00-0.25], p = 0.05). That is, a 1SD advantage in childhood cognitive ability predicted 0.13 more deaths per increase in father's social class. Note that this association was much smaller in magnitude than the decreases in additional deaths associated with the main effects.

Additive models were constructed to examine the IQ-mortality association within each social class, including those whose father's occupational social class was missing, as in the main text (Table A4). The direction and pattern of associations was consistent with the Cox Proportional Hazards models used in the main text: a 1SD advantage in IQ was significantly associated with fewer deaths per person year among those from unskilled, semi-skilled and skilled social class backgrounds. The benefit of advantage in IQ appeared to diminish slightly as class increased, suggesting that high IQ may benefit those from lower social classes most. Note again, however, that the interaction effect in the whole-sample model above was not significant. There was no significant association between IQ and mortality in those from intermediate, professional or missing occupational social classes, and confidence intervals in these groups were wide.

We conclude that the additive hazards approach presented here is in keeping with the proportional hazards approach used in the main text. In the context of the underlying mortality hazard for this sample, advantage in IQ and childhood social class predict modest reductions in the number of deaths per 10,000 person years. In contrast, the interaction effect was relatively weak, with no consistent trend across classes.

Iveson MH, *et al. BMJ Open* 2020; 10:e037847. doi: 10.1136/bmjopen-2020-037847

Table A3. Results from weighted Additive Hazards regression models predicting all-cause mortality risk. Additional deaths per 10,000 person years and 95% Confidence Intervals for IQ score and Father's social class in univariate models, models adjusted for sex and age at Scottish Mental Survey 1947, models adjusted for both IQ score and Father's social class (and sex and age), and a model additionally including the IQ-Social class interaction effect (and age and sex).

	Univariate		Sex and age-adjusted		Mutually-adjusted	
**Predictor**	**Additional deaths per 10,000 person years [95% CI]**	**p**	**Additional deaths per 10,000 person years [95% CI]**	**p**	**Additional deaths per 10,000 person years [95% CI]**	**p**
IQ score (1 SD)	-0.57 [-0.69, -0.45]	<0.001	-0.56 [-0.68, -0.44]	<0.001	-0.53 [-0.66, -0.41]	<0.001
Father's social class (1 class)	-0.31 [-0.43, -0.19]	<0.001	-0.31 [-0.43, -0.19]	<0.001	-0.20 [-0.33, -0.08]	<0.01

Table A4. Additional deaths per 10,000 person years and 95% Confidence Intervals for IQ score, adjusted for sex and age at Scottish Mental Survey 1947, in each of the Father's social classes.

	**Additional deaths per 10,000 person years [95% CI]**	**p**
Unskilled (N = 1073)	-3.37 [-4.58, -2.15]	<0.001
Semi-skilled (N = 1026)	-2.67 [-4.09, -1.24]	<0.001
Skilled (N = 2701)	-1.05 [-1.41, -0.68]	<0.001
Intermediate (N = 472)	-3.62 [-8.26, 1.02]	0.13
Professional (N = 46)	31.16 [-146.18, 208.50]	0.73
Missing social class (N = 109)[1]	-42.56 [-88.32, 3.19]	0.07

[1]Includes individuals with missing father's social class but with complete MHT scores and vitality status.

References

1. VanderWeele TJ, Ding P. Sensitivity analysis in observational research: Introducing the E-value. Annals of Internal Medicine. 2017;167:268-274. doi: 10.7326/M16-2607
2. Rod NJ, Lange T, Andersen I, Marott JL, Diderichsen F. Additive interaction in survival analysis: Use of the additive hazards model. Epidemiology. 2012;23(5):733-737.
