# Supplementary material for: Do childhood socioeconomic circumstances moderate the association between childhood cognitive ability and all-cause mortality across the life course? Prospective observational study of the 36-day sample of the Scottish Mental Survey 1947
Source: BMJ Open. 2020 Dec 2;10(12):e037847. doi: 10.1136/bmjopen-2020-037847 (PMC7712420; doi:10.1136/bmjopen-2020-037847)
Supplement: Author's manuscript [file bmjopen-2020-037847.draft_revisions.pdf]

BMJ Open is committed to open peer review. As part of this commitment we make the peer review history of every article we publish publicly available.

When an article is published we post the peer reviewers' comments and the authors' responses online. We also post the versions of the paper that were used during peer review. These are the versions that the peer review comments apply to.

The versions of the paper that follow are the versions that were submitted during the peer review process. They are not the versions of record or the final published versions. They should not be cited or distributed as the published version of this manuscript.

BMJ Open is an open access journal and the full, final, typeset and author-corrected version of record of the manuscript is available on our site with no access controls, subscription charges or pay-per-view fees (http://bmjopen.bmj.com).

If you have any questions on BMJ Open's open peer review process please email info.bmjopen@bmj.com

BMJ Open

Do childhood socioeconomic circumstances moderate the association between childhood cognitive ability and all-cause mortality?

Journal:	*BMJ Open*
Manuscript ID	bmjopen-2020-037847
Article Type:	Original research
Date Submitted by the Author:	18-Feb-2020

Complete List of Authors:	Iveson, Matthew; University of Edinburgh, Centre for Clinical Brain Sciences<br>Altschul, Drew; University of Edinburgh, Department of Psychology<br>Deary, Ian; University of Edinburgh, Department of Psychology

Keywords:	EPIDEMIOLOGY, PUBLIC HEALTH, STATISTICS & RESEARCH METHODS

SCHOLARONE™
Manuscripts

I, the Submitting Author has the right to grant and does grant on behalf of all authors of the Work (as defined in the below author licence), an exclusive licence and/or a non-exclusive licence for contributions from authors who are: i) UK Crown employees; ii) where BMJ has agreed a CC-BY licence shall apply, and/or iii) in accordance with the terms applicable for US Federal Government officers or employees acting as part of their official duties; on a worldwide, perpetual, irrevocable, royalty-free basis to BMJ Publishing Group Ltd ("BMJ") its licensees and where the relevant Journal is co-owned by BMJ to the co-owners of the Journal, to publish the Work in this journal and any other BMJ products and to exploit all rights, as set out in our licence.

The Submitting Author accepts and understands that any supply made under these terms is made by BMJ to the Submitting Author unless you are acting as an employee on behalf of your employer or a postgraduate student of an affiliated institution which is paying any applicable article publishing charge ("APC") for Open Access articles. Where the Submitting Author wishes to make the Work available on an Open Access basis (and intends to pay the relevant APC), the terms of reuse of such Open Access shall be governed by a Creative Commons licence – details of these licences and which Creative Commons licence will apply to this Work are set out in our licence referred to above.

Other than as permitted in any relevant BMJ Author's Self Archiving Policies, I confirm this Work has not been accepted for publication elsewhere, is not being considered for publication elsewhere and does not duplicate material already published. I confirm all authors consent to publication of this Work and authorise the granting of this licence.

For peer review only

Do childhood socioeconomic circumstances moderate the association between childhood cognitive ability and all-cause mortality?

Matthew H Iveson*[1,2], Drew Altschul[1,2], and Ian J Deary[2,3]

[1]MRC Mental Health Data Pathfinder team, The University of Edinburgh, Edinburgh, UK; [2]The Centre for Cognitive Ageing and Cognitive Epidemiology, Department of Psychology, The University of Edinburgh, Edinburgh, UK; [3]Lothian Birth Cohorts, Department of Psychology, The University of Edinburgh, Edinburgh, UK.

Word count: 2789

Correspondence to:

Dr Matthew H. Iveson, Department of Psychology, The University of Edinburgh, 7 George Square, Edinburgh, EH8 9JZ, United Kingdom

For peer review only - http://bmjopen.bmj.com/site/about/guidelines.xhtml

Abstract

Background

There is growing evidence that higher childhood cognitive ability predicts lower all-cause mortality risk across the life course. Whereas this association does not appear to be mediated by childhood socioeconomic circumstances, it is unclear whether socioeconomic circumstances moderate this association.

Methods

The moderating role of childhood socioeconomic circumstances was assessed in 5318 members of the 36-day sample of the Scottish Mental Survey 1947. Univariate, sex-adjusted, and mutually-adjusted models predicting all-cause mortality risk up to age 79 were created using childhood IQ scores and childhood social class as predictors. Moderation was assessed by adding an interaction term between IQ scores and social class and comparing model fit.

Results

A standard deviation advantage in childhood IQ scores [Hazard Ratio (HR) = 0.83, 95% Confidence Interval (CI) = 0.79-0.86, $p < 0.001$] and a single class advantage in childhood social class [HR = 0.92, 95% CI = 0.88-0.97, $p < 0.001$] independently predicted lower mortality risk. Adding the IQ-social class interaction effect did not improve model fit [$X^2\Delta = 1.28$, $p = 0.26$], and the interaction effect did not predict mortality risk [HR = 1.03, 95% CI = 0.97-1.07, $p = 0.257$].

Conclusions

The present study demonstrated that the association between higher childhood cognitive ability and lower all-cause mortality risk is not conditional upon childhood social class. Whereas other measures of socioeconomic circumstances may play a moderating role, these findings suggest that the benefits of higher childhood cognitive ability for longevity apply regardless of the material socioeconomic circumstances experienced in childhood.

For peer review only - http://bmjopen.bmj.com/site/about/guidelines.xhtml

For peer review only

Keywords:

Mortality; Intelligence; Socioeconomic circumstances; Moderation effects

Article Summary

Strengths and limitations of this study

- All-cause mortality risk was examined in a large, nationally-representative sample over an entire life course.
- The study used contemporaneously-measured indicators of both childhood socioeconomic circumstances and childhood cognitive ability.
- The present study used a more fine-grained measure of childhood socioeconomic circumstances than previous research that better captures material resources in early life.
- Use of parental social class as a measure of childhood socioeconomic circumstances means potential miscoding of those whose parents were not employed.
- The particular social, economic and educational changes experienced by these older adults may limit the generalisability to younger cohorts.

For peer review only

For peer review only - http://bmjopen.bmj.com/site/about/guidelines.xhtml

Introduction

Several studies have demonstrated an association between higher cognitive ability and lower risk of all-cause mortality (1–3). This association stretches across the life-course, with childhood cognitive ability predicting mortality risk up to much later in life (4–8). There have been several attempts to model these associations while accounting for a suite of demographic and psychosocial factors as covariates. Perhaps being the most common covariate to be examined, some studies have reported that childhood socioeconomic circumstances mediate or confound the association between early-life cognitive ability and all-cause mortality risk either very slightly or not at all (6,7,9–11). However, the commonality between these studies has been the tendency to focus on examining only confounding and mediation effects while ignoring potential moderation of the cognitive ability-mortality association by socioeconomic circumstances. Whereas a mediator explains the association between two other variables (i.e., between childhood cognitive ability and all-cause mortality), a moderator influences the strength of the association between the variables. That is, the strength of the association between childhood cognitive ability and all-cause mortality risk may be conditional on socioeconomic circumstances; a strong association may be observed for some levels of socioeconomic circumstances, but a weaker (or no) association may be observed for others.

Only a few studies have considered the moderating role of socioeconomic circumstances in the association between cognitive ability and all-cause mortality. In a study using the Helsinki Birth Cohort, higher cognitive ability at age 20-years was associated with lower risk of mortality in later-life, and this association was stronger among those from middle-class than manual-occupational-class families (12). Similar moderation has been shown in the US National Longitudinal Survey of Youth 1979, albeit using parental education instead of occupational class (13). In that study, the association between higher cognitive ability in young adulthood and lower all-cause mortality risk was significant only for those whose parents had high school qualifications or above, and not for those whose parents had not completed high school. However, these two studies are limited in

For peer review only

terms of their samples – such as examining only males (12) – and in terms of their mortality follow-up – such as covering only mid-life mortality (13). Furthermore, these studies use early adulthood cognitive ability as a predictor of mortality, whereas many studies report a strong association between cognitive ability in childhood and mortality across the life course (4–7). Although there appears to be little evidence that childhood socioeconomic circumstances mediate the association between childhood cognitive ability and all-cause mortality risk, their role as a moderator of this association remains unclear.

In the present study we examine the role of childhood socioeconomic circumstances as a possible moderator of the association between childhood cognitive ability and all-cause mortality risk across the life course. In particular, we utilise a representative sample of men and women born in Scotland in 1936 and whose cognitive ability was assessed as part of the Scottish Mental Survey 1947 and who were followed for mortality up to 2015. Importantly, an association between childhood cognitive ability and all-cause mortality has already been established in this sample (HR = 0.80, 95% CI [0.78, 0.81]; 6, 14) and in a subsample of these individuals (HR = 0.76, 95% CI [0.68, 0.84]; 7); however, potential moderation by childhood socioeconomic circumstances has not been investigated. Given previous observations using adult cognitive ability (12,13), we predicted that childhood socioeconomic circumstances would significantly moderate the association between childhood cognitive ability and all-cause mortality, with stronger associations in those whose fathers had less manual-based occupations.

Methods

Sample

The sample consisted of 6291 individuals from the 36-day sample of the Scottish Mental Survey 1947 (15). These individuals, born on one of 36 birth dates in 1936 (the first three days of each month),

For peer review only - http://bmjopen.bmj.com/site/about/guidelines.xhtml

took part in a nationwide test of intelligence on the 4th of June 1947 (age 11). They were subsequently followed-up across the life course, both in person and remotely using routinely-collected data (15–17). In particular, these individuals were traced in national migration and death records.

To create the analytic sample (N = 5318), we removed individuals with missing childhood cognitive ability (N = 575), missing childhood socioeconomic circumstances (N = 130), or missing date of death (if deceased, N = 13). Note that these individuals could have more than one key variable missing. Individuals who emigrated after completing the Scottish Mental Survey 1947 but before the end of the surveillance period were retained and censored appropriately. The study was approved by the local ethics and conformed to the Declaration of Helsinki.

For peer review only

Measures

Cognitive ability

Childhood cognitive ability around age 11 years was assessed using the Moray House Test No. 12 test of intelligence (MHT; 15) administered as part of the Scottish Mental Survey 1947. The MHT consists of 71 items testing verbal reasoning, arithmetic, spatial problem-solving, and so on. Further details of the MHT and its administration are provided elsewhere (18). Raw MHT scores (ranging from 0 to 76) were converted to IQ-type score (Mean = 100, SD = 15) and then standardised (z-transformed).

Socioeconomic circumstances

The occupational social class of the father was used as a proxy of childhood socioeconomic circumstances at age 11 years. Father's occupation was recorded in 1947 as part of the Sociological Schedule – a planned follow-up to the Scottish Mental Survey 1947 (15). Father's occupation was

For peer review only - http://bmjopen.bmj.com/site/about/guidelines.xhtml

later categorised into one of 5 occupational social classes according to the 1950 United Kingdom's classification index (19). Note that this was based on their current or most recent occupation; fathers who were without occupations or who were long-term unemployed were coded as missing. The social classes were reversed so that higher-numbered classes represented more professional occupations: 1) Unskilled, 2) Partly-skilled, 3) Skilled, 4) Managerial, 5) Professional.

Mortality

All-cause mortality (status and date of death) was obtained from National Records of Scotland death records covering 1947 to 2015. The linkage and extraction of death records have been described in detail elsewhere (7,16). Individuals were censored at the end of mortality surveillance (30th[th] November 2015). Survival time was calculated as the number of days between birth and death or censoring date, as appropriate.

<u>Analyses</u>

We first described the demographic characteristics of the analytic sample, split by mortality status, before examining cohort selection effects by comparing excluded and retained individuals.

We then conducted survival analyses using Cox proportional hazards regression. Hazard ratios were estimated to indicate the proportional change in all-cause mortality risk associated with a unit change in each predictor. As in previous work, individual models were constructed to assess the association between standardised IQ scores and reversed father's occupational social class (separately) and survival odds. Models were then adjusted for sex. A mutually-adjusted model was then created – including cognitive ability, social class and sex. Novel to this study, we further constructed a model additionally including an interaction term between cognitive ability and social class. In addition to examining predictors within a given model, the effect of adding a given predictor

For peer review only - http://bmjopen.bmj.com/site/about/guidelines.xhtml

For peer review only

was assessed by comparing the fit of successive models (e.g., mutually-adjusted vs mutually-adjusted and moderated) using Chi-squared tests. Univariate survival models including standardised IQ score were then created for each social class in order to better assess interaction effects (or lack thereof).

Analysis was conducted in R (20) using RStudio (21), and with the 'psych' (22) and 'survival' (23) packages.

The analysis plan was preregistered prior to the data being analysed (https://osf.io/u8y9n/). The only change to the planned analysis was the treatment of father's occupational social class as numeric (not centred or transformed) during the survival analyses, rather than ordinal. This was done because use of the ordinal variable violated the proportional hazards assumption, and use of a numeric variable provided similar model fit to a categorical variable.

For peer review only

Patient and public involvement

This study was based on analysis of previously collected and anonymised research data and routinely-collected health data. As such, neither participants nor the public were involved in the design or planning of this study.

Results

Descriptive statistics for the analytic sample, split by father's occupational social class, are shown in Table 1. Mortality status ($p < 0.001$), survival times ($p < 0.001$) and MHT scores ($p < 0.001$) significantly differed between social classes, with a higher proportion of those in higher social classes being alive at censor date, surviving longer, and demonstrating higher MHT scores. Note, however, that the mean survival time of individuals with fathers from skilled occupations was lower than those with fathers from semi-skilled occupations, despite a lower proportion of deceased

For peer review only - http://bmjopen.bmj.com/site/about/guidelines.xhtml

individuals. Furthermore, there were relatively few individuals with a father from a professional occupation. MHT scores were significantly and positively correlated with both father's occupational social class (Spearman's rho = 0.19, $p < 0.001$) and survival time (Spearman's rho = 0.11, $p < 0.001$). Likewise, social class was significantly and positively correlated with survival time (Spearman's rho = 0.04, $p < 0.01$).

For peer review only

For peer review only - http://bmjopen.bmj.com/site/about/guidelines.xhtml

Table 1. Descriptive statistics for the analytic sample, split by father's occupational social class.

	Unskilled (N = 1073)	Semi-skilled (N = 1026)	Skilled (N = 2701)	Intermediate (N = 472)	Professional (N = 46)	Total (N = 5318)
**Sex**						
N (%) Male	546 (50.9%)	530 (51.7%)	1332 (49.3%)	239 (50.6%)	21 (45.7%)	2668 (50.2%)
N (%) Female	527 (49.1%)	496 (48.3%)	1369 (50.7%)	233 (49.4%)	25 (54.3%)	2650 (49.8%)
**Moray House Test Score (max = 76)**						
Mean (SD)	31.88 (15.39)	33.67 (15.57)	38.21 (15.18)	39.74 (15.91)	48.85 (12.69)	36.29 (15.65)
**Mortality status**						
N (%) Alive	597 (55.6%)	632 (61.6%)	1703 (63.1%)	321 (68.0%)	32 (69.6%)	3285 (61.8%)
N (%) Dead	476 (44.4%)	394 (38.4%)	998 (36.9%)	151 (32.0%)	14 (30.4%)	2033 (38.2%)
**Time from birth to death/censor (years)**						
Mean (SD)	68.11 (16.82)	68.75 (17.01)	67.76 (18.15)	70.93 (15.65)	71.83 (16.63)	68.34 (17.46)

For peer review only

We then examined selection effects by comparing MHT scores and social class between the analytic sample and those removed due to having missing key variables. Removed individuals had significantly lower MHT score (removed mean = 34.4, $t(454) = -2.22$, $p < 0.05$), but the samples did not significantly differ in terms of social class ($X^2 = 9.30$, $p = 0.054$). This trend has been reported elsewhere (24,25) and may indicate that individuals with lower childhood cognitive ability were more likely to have long-term unemployed fathers who could not be ascribed an occupational social class. Note, however, that differences in MHT score were small; removed individuals scored on average 1.87 points fewer on the MHT.

Returning to the analytic sample, we next compared descriptive statistics between those alive (N = 3285) and those dead (N = 2033) (Table 1). In terms of sex, proportionately more males had died than females ($p < 0.001$). Those who died exhibited significantly lower MHT scores (M = 33.40, SD = 15.89) and tended to be from more manual social classes (Unskilled = 23.4%) than those who survived (MHT: M = 38.07, SD = 15.23, $p < 0.001$; Social class: Unskilled = 18.2%, $p < 0.001$).

We then conducted survival analyses aimed at examining whether social class moderated the association between childhood cognitive ability and all-cause mortality risk. Results from the Schoenfeld test and visual inspection of the scaled Schoenfeld residuals supported the proportional hazard assumption for all predictors in each model (once ordinal social class was replaced with numeric social class). Kaplan-Meier curves showing the association between IQ and survival within each social class are shown in Figure 1.

--Insert Figure 1 here--

Before examining moderation effects, we tested the independent associations of age-11 IQ and father's social class with all-cause mortality risk, as in previous work. In the univariate models, a 1SD

For peer review only - http://bmjopen.bmj.com/site/about/guidelines.xhtml

advantage in age-11 IQ predicted a 19% lower mortality risk over time (Table 2). A 1-class advantage in father's social class predicted an 11% lower mortality risk over time. The strength of these associations was similar after adjusting for sex, though adding sex significantly improved the fit of each model (ps < 0.001). Likewise, mutual-adjustment (including sex) did not wholly or partly attenuate any one association with mortality risk, and significantly improved model fit versus both sex-adjusted models (ps < 0.001).

For peer review only

For peer review only - http://bmjopen.bmj.com/site/about/guidelines.xhtml

Table 2. Regression coefficients and hazard ratios (HR) for IQ score and Father's social class in univariate models, models adjusted for sex, models adjusted for both IQ score and Father's social class (and sex), and a model including an interaction term (and sex).

	Univariate[1,2]				Sex-adjusted[3,4]				Mutually-adjusted[5]				Interaction[6]			
**Predictor**	**B**	**SE**	**HR [95% CI]**	**p**	**B**	**SE**	**HR [95% CI]**	**p**	**B**	**SE**	**HR [95% CI]**	**p**	**B**	**SE**	**HR [95% CI]**	**p**
IQ score (1 SD)	-0.21	0.02	0.81 [0.77-0.84]	<0.001	-0.20	0.02	0.82 [0.78-0.85]	<0.001	-0.19	0.02	0.83 [0.79-0.86]	<0.001	-0.25	0.06	0.78 [0.69-0.87]	<0.001
Father's social class (1 class)	-0.12	0.02	0.89 [0.85-0.93]	<0.001	-0.12	0.02	0.89 [0.85-0.93]	<0.001	-0.08	0.02	0.92 [0.88-0.97]	<0.001	-0.07	0.02	0.93 [0.89-0.97]	0.002
IQ X Social class													0.03	0.02	1.03 [0.97-1.07]	0.257

[1]Univariate IQ model R^2 = 0.018; [2]Univariate Father's social class model R^2 = 0.005; [3]Sex-adjusted IQ model R^2 = 0.033; [4]Sex-adjusted Father's social class model R^2 = 0.022; [5]Mutually-adjusted model R^2 = 0.035; [6]Interaction model R^2 = 0.035.

For peer review only

For peer review only - http://bmjopen.bmj.com/site/about/guidelines.xhtml

We then tested the moderating effect of father's social class. Adding the interaction term (age-11 IQ by father's social class) did not significantly improve model fit versus the mutually-adjusted model (Mutually-adjusted AIC = 33230.83, Interaction AIC = 33231.55; $X^2\Delta = 1.28$, $p = 0.26$). The interaction effect did not significantly predict all-cause mortality risk. Adding the interaction term further introduced a multicollinearity issue (VIF = 7.22) – strong collinearity between predictors (sex, father's social class, age-11 IQ and the interaction term) indicating some redundancy – which was not present in the mutually-adjusted model. In the interaction model, a 1SD advantage in age-11 IQ and a 1-class advantage in father's social class both independently predicted lower mortality risk (22% and 7%, respectively) (Table 2).

Finally, we examined the association between age-11 IQ and all-cause mortality risk in each of the social classes (Table 3). A 1SD advantage in age-11 IQ significantly predicted reduced mortality risk among those from unskilled (21% decrease), semi-skilled (17% decrease) and skilled (17% decrease) backgrounds. The same direction of association, albeit not statistically significant, was observed among those from intermediate backgrounds. There was also no significant association between age-11 IQ and mortality risk among those from professional backgrounds, though this group was notably small (N = 46).

Table 3. Regression coefficients and hazard ratios (HR) for IQ score, adjusted for sex, in each of the Father's social classes.

	**B**	**SE**	**HR [95% CI]**	**p**	**R^2**	**AIC**
Unskilled (N = 1073)	-0.24	0.05	0.79 [0.72-0.86]	<0.001	0.045	6232.63
Semi-skilled (N = 1026)	-0.19	0.05	0.83 [0.75-0.91]	<0.001	0.023	5161.63
Skilled (N = 2701)	-0.19	0.03	0.83 [0.78-0.88]	<0.001	0.031	14947.84
Intermediate (N = 472)	-0.13	0.08	0.88 [0.75-1.02]	0.099	0.031	1768.52
Professional (N = 46)	0.06	0.36	1.06 [0.52-2.16]	0.866	0.001	104.52

For peer review only - http://bmjopen.bmj.com/site/about/guidelines.xhtml

Discussion

The present study aimed to establish whether the association between higher childhood cognitive ability and reduced all-cause mortality risk is moderated by childhood socioeconomic circumstances. Whereas advantage in terms of childhood cognitive ability and father's social class independently predicted reduced risk of all-cause mortality, there was no evidence of a significant interaction effect. This supports previous observations that advantage in cognitive ability and socioeconomic circumstances have separate contributions to lower all-cause mortality risk (6,7), and further suggests that the benefits of high cognitive ability apply regardless of social class.

The lack of a significant moderation effect by socioeconomic circumstances is contrary to the few previous studies to examine this (12,13). Notably, Jokela et al. (13) observed a significant (albeit small) moderating effect of parental education, with a stronger IQ-mortality association among those with more educated parents, rather than occupational social class as used here. Whereas parental education is thought to reflect the intellectual resources of the family, parental occupation is more indicative of the material resources of the family (26). The benefits of higher cognitive ability for health, therefore, may not depend on the material resources available in childhood. Whereas Kajantie et al. (12) did observe a significant moderation effect using parental occupational social class, only two categories were used (manual and middle-class). Such a coarse measure of occupational social class may overestimate its contribution to all-cause mortality risk. At the same time, occupational social class cannot be coded for those who are unemployed or retired. This may result in a misrepresentation of the importance of childhood socioeconomic circumstances both in Kajantie et al. (12) and in the present study.

The main strength of the present study is the use of both socioeconomic circumstances and cognitive ability measured contemporaneously in childhood. Childhood cognitive ability and childhood socioeconomic circumstances have been shown to predict later-life health, even after accounting for socioeconomic circumstances in adulthood (5,6,25,27). Measuring these factors

For peer review only - http://bmjopen.bmj.com/site/about/guidelines.xhtml

contemporaneously avoids potential recall bias associated with retrospective measures of socioeconomic circumstances in particular (28). Also, as individuals must survive until cognitive and socioeconomic measures are taken, examining these factors in childhood lessens potential selection effects (e.g., lower cognitive ability individuals who may die prior to adulthood). Furthermore, the present study examined all-cause mortality risk in a nationally-representative sample over an entire life course (age 11-79 years-old). These individuals have had longer exposure to mortality risk than the younger samples used in previous work (12,13).

A limitation is that the sample is a product of the time and place in which they lived (29), and this may limit the generalisability of the results. All individuals in the sample were born in 1936 and lived in Scotland in 1947. They survived the Second World War and lived through the significant social, economic and educational changes that followed. Childhood socioeconomic conditions (including occupational and educational opportunities of parents) in more modern samples are likely better overall than those captured in the present sample (30). Indeed, relatively few individuals originated from higher childhood social classes (intermediate or professional occupations). Similarly, the meaning of occupational social class is likely to have changed as new occupations are created and working conditions improved (31). Furthermore, these individuals had access to universal healthcare throughout their lives, likely reducing the importance of socioeconomic circumstances as both a predictor of mortality (32) and a moderator of the cognitive ability-mortality association.

Consistent with previous work, the present study demonstrates an association between higher cognitive ability and lower all-cause mortality risk. However, it also suggests that this association is not conditional on one's social class of origin. Whereas childhood social class was an important independent predictor of all-cause mortality risk, there was no evidence that it moderated the role of childhood cognitive ability in predicting longevity. The benefits of higher childhood cognitive ability for lower all-cause mortality risk were observed across childhood social classes. This is an important consideration for public health policy and interventions: childhood cognitive ability is a

For peer review only - http://bmjopen.bmj.com/site/about/guidelines.xhtml

For peer review only

source of health inequality independent from material socioeconomic resources in childhood, and needs to be addressed separately. This being said, it is unclear whether other measures of childhood socioeconomic circumstances, such as parental education, play a role in moderating the benefits of higher childhood cognitive ability. Future work should focus on clarifying the mechanisms by which childhood cognitive ability and childhood socioeconomic conditions affect mortality risk.

Contributors

All authors contributed to the conceptualization and design of this study. MHI contributed to data curation and formal analysis. MHI, DA and IJD contributed to investigation, methodology, project administration, validation, visualisation, writing – original draft preparation, and writing – review & editing. IJD contributed to funding acquisition and supervision.

Data sharing

Historical data from the Scottish Mental Survey 1947 is available upon request from the Department of Psychology, The University of Edinburgh. Access is contingent on local governance approvals. Routinely-collected records, including national death records, are available and linkable through NHS Information Services Division and the eData Research and Innovation Service (https://www.isdscotland.org/Products-and-Services/EDRIS/). Access is contingent on ethical and Public Benefit and Privacy Panel approvals.

Ethics statement

For peer review only - http://bmjopen.bmj.com/site/about/guidelines.xhtml

For peer review only

This submission is covered by the ethics application "Lifelong health and wellbeing of the 'Scotland in Miniature' cohort: The 6-Day Sample of the Scottish Mental Survey" (REC# 12/SS/0024; Scotland A Research Ethics Committee).

Funding statement

This work was supported by a Medical Research Council Mental Health Data Pathfinder award [MRC-MC_PC_17209], and by a UK cross council Lifelong Health and Wellbeing Initiative [MRCG1001401], for which IJD is the principal investigator. MHI, DA and IJD are members of The University of Edinburgh Centre for Cognitive Ageing and Cognitive Epidemiology, part of the cross council Lifelong Health and Wellbeing Initiative [MR/K026992/1].

For peer review only

References

1. Korten AE, Jorm AF, Jiao Z, Letenneur L, Jacomb PA, Henderson AS, et al. Health, cognitive, and psychosocial factors as predictors of mortality in an elderly community sample. J Epidemiol Community Health. 1999;53(2):83–8.

2. Pavlik VN, De Moraes SA, Szklo M, Knopman DS, Mosley TH, Hymans DJ. Relation between cognitive function and mortality in middle-aged adults: The atherosclerosis risk in communities study. Am J Epidemiol. 2003;157(4):327–34.

3. Batterham PJ, Christensen H, Mackinnon AJ. Fluid intelligence is independently associated with all-cause mortality over 17 years in an elderly community sample: An investigation of potential mechanisms. Intelligence [Internet]. 2009;37(6):551–60. Available from: http://dx.doi.org/10.1016/j.intell.2008.10.004

4. Bratsberg B, Rogeberg O. Childhood socioeconomic status does not explain the IQ-mortality gradient. Intelligence [Internet]. 2017;62(March):148–54. Available from: http://dx.doi.org/10.1016/j.intell.2017.04.002

5. Calvin CM, Deary IJ, Fenton C, Roberts BA, Der G, Leckenby N, et al. Intelligence in youth and all-cause-mortality: Systematic review with meta-analysis. Int J Epidemiol. 2011;40(3):626–44.

6. Calvin CM, Batty GD, Der G, Brett CE, Taylor A, Pattie A, et al. Childhood intelligence in relation to major causes of death in 68 year follow-up: Prospective population study. BMJ. 2017;357.

7. Iveson MH, Čukič I, Der G, Batty GD, Deary IJ. Intelligence and all-cause mortality in the 6-Day Sample of the Scottish Mental Survey 1947 and their siblings: Testing the contribution of family background. Int J Epidemiol. 2018;47(1).

8. Whalley LJ, Deary IJ. Longitudinal cohort study of childhood IQ and survival up to age 76. Br

For peer review only - http://bmjopen.bmj.com/site/about/guidelines.xhtml

Med J. 2001;322(7290):819–22.

9. Kuh D, Richards M, Hardy R, Butterworth S, Wadsworth MEJ. Childhood cognitive ability and deaths up until middle age: A post-war birth cohort study. Int J Epidemiol. 2004;33(2):408–13.

10. Batty GD, Wennerstad KM, Smith GD, Gunnell D, Deary IJ, Tynelius P, et al. IQ in early adulthood and mortality by middle age: Cohort study of 1 million swedish men. Epidemiology. 2009;20(1):100–9.

11. Lager A, Bremberg S, Vågerö D. The association of early IQ and education with mortality: 65 Year longitudinal study in Malmö, Sweden. BMJ. 2009;339(7735):1432.

12. Kajantie E, Räikkönen K, Henriksson M, Forsén T, Heinonen K, Pesonen AK, et al. Childhood socioeconomic status modifies the association between intellectual abilities at age 20 and mortality in later life. J Epidemiol Community Health. 2010;64(11):963–9.

13. Jokela M, Elovainio M, Singh-Manoux A, Kivimäki M. IQ, Socioeconomic status, and early death: The US national longitudinal survey of youth. Psychosom Med. 2009;71(3):322–8.

14. Čukić I, Brett CE, Calvin CM, Batty GD, Deary IJ. Childhood IQ and survival to 79: Follow-up of 94% of the Scottish Mental Survey 1947. Intelligence. 2017;63(May):45–50.

15. The Scottish Council for Research in Education. The trend of Scottish intelligence. London: University of London Press; 1949.

16. Brett CE, Deary IJ. Realising health data linkage from a researcher's perspective: following up the 6-Day Sample of the Scottish Mental Survey 1947. Longit Life Course Stud. 2014;5(3):283–98.

17. Deary IJ, Brett CE. Predicting and retrodicting intelligence between childhood and old age in the 6-Day Sample of the Scottish Mental Survey 1947. Intelligence [Internet]. 2015;50:1–9.

For peer review only

For peer review only - http://bmjopen.bmj.com/site/about/guidelines.xhtml

Available from: http://dx.doi.org/10.1016/j.intell.2015.02.002

18. Deary IJ, Whiteman MC, Starr JM, Whalley LJ, Fox HC. The Impact of Childhood Intelligence on Later Life: Following Up the Scottish Mental Surveys of 1932 and 1947. J Pers Soc Psychol. 2004;86(1):130–47.

19. General Register Office. Census, 1951: Classification of Occupations. London: HMSO; 1956.

20. R Core Team. R: A language and environment for statistical computing. Vienna: R Foundation for Statistical Computing; 2018.

21. RStudio Team. RStudio: Integrated development for R. Boston, MA: RStudio Inc.; 2016.

22. Revelle W. psych: Procedures for personality and psychological research. Evanston: Northwestern University; 2018.

23. Therneau TM, Lumley T. Package "survival." R Top Doc. 2015;128.

24. Johnson W, Brett CE, Calvin C, Deary IJ. Childhood characteristics and participation in Scottish Mental Survey 1947 6-Day Sample Follow-ups: Implications for participation in aging studies. Intelligence [Internet]. 2016;54:70–9. Available from: http://dx.doi.org/10.1016/j.intell.2015.11.006

25. Iveson MH, Deary IJ. Intergenerational social mobility and subjective wellbeing in later life. Soc Sci Med [Internet]. 2017;188:11–20. Available from: http://dx.doi.org/10.1016/j.socscimed.2017.06.038

26. Galobardes B, Shaw M, Lawlor DA, Lynch JW, Smith GD. Indicators of socioeconomic position (part 1). J Epidemiol Community Health. 2006;60(1):7–12.

27. Iveson MH, Deary IJ. Early-life predictors of retirement decisions and post-retirement health. SSM - Popul Heal [Internet]. 2019;8(February):100430. Available from: https://doi.org/10.1016/j.ssmph.2019.100430

For peer review only

For peer review only - http://bmjopen.bmj.com/site/about/guidelines.xhtml

28. Batty GD, Lawlor DA, Macintyre S, Clark H, Leon DA. Accuracy of adults' recall of childhood social class: Findings from the Aberdeen children of the 1950s study. J Epidemiol Community Health. 2005;59(10):898–903.

29. Elder Jr GH. Time, human agency, and social change: Perspectives on the life course. Soc Psychol Q. 1994;4–15.

30. Galobardes B, Lynch JW, Smith GD. Childhood socioeconomic circumstances and cause-specific mortality in adulthood: Systematic review and interpretation. Epidemiol Rev. 2004;26:7–21.

31. Goldthorpe JH. On Economic Development and Social Mobility. Br J Soc. 1985;36(4):549–73.

32. Asaria M, Ali S, Doran T, Ferguson B, Fleetcroft R, Goddard M, et al. How a universal health system reduces inequalities: Lessons from England. J Epidemiol Community Health. 2016;70(7):637–43.

For peer review only

For peer review only - http://bmjopen.bmj.com/site/about/guidelines.xhtml

Figure 1. Kaplan-Meier Survival curves showing survival probability associated with IQ z-scores 1 SD below (red), around (green) or above (blue) the mean, split by father's occupational social class.

For peer review only

For peer review only - http://bmjopen.bmj.com/site/about/guidelines.xhtml

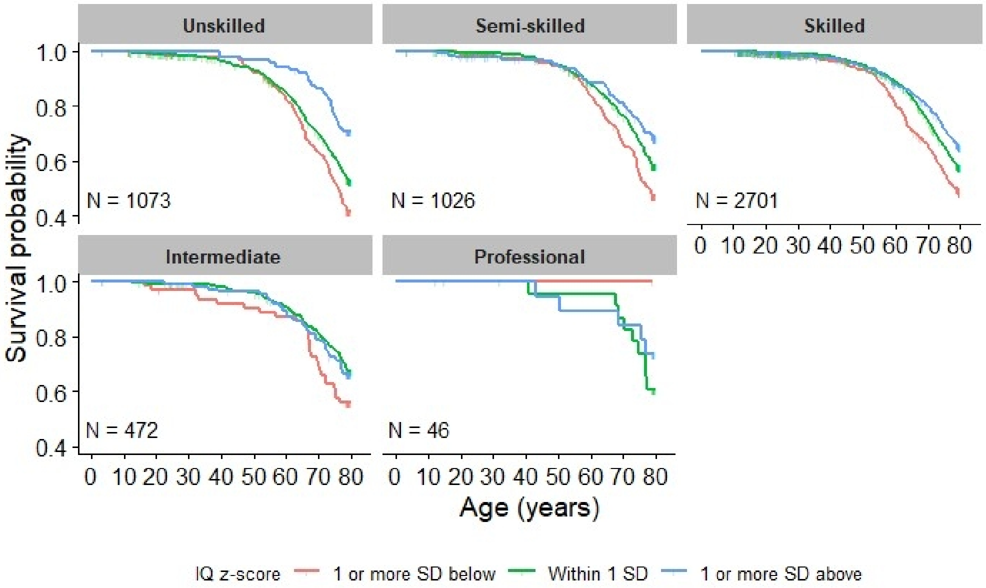

185x114mm (96 x 96 DPI)

Do childhood socioeconomic circumstances moderate the association between childhood cognitive ability and all-cause mortality across the life course? Prospective observational study of the 36-Day Sample of the Scottish Mental Survey 1947.

Journal:	*BMJ Open*
Manuscript ID	bmjopen-2020-037847.R1
Article Type:	Original research
Date Submitted by the Author:	11-Aug-2020
Complete List of Authors:	Iveson, Matthew; The University of Edinburgh, Centre for Clinical Brain Sciences<br>Altschul, Drew; The University of Edinburgh, Department of Psychology<br>Deary, Ian; The University of Edinburgh, Department of Psychology
**Primary Subject Heading**:	Epidemiology
Secondary Subject Heading:	Epidemiology, Public health
Keywords:	EPIDEMIOLOGY, PUBLIC HEALTH, STATISTICS & RESEARCH METHODS

SCHOLARONE™
Manuscripts

I, the Submitting Author has the right to grant and does grant on behalf of all authors of the Work (as defined in the below author licence), an exclusive licence and/or a non-exclusive licence for contributions from authors who are: i) UK Crown employees; ii) where BMJ has agreed a CC-BY licence shall apply, and/or iii) in accordance with the terms applicable for US Federal Government officers or employees acting as part of their official duties; on a worldwide, perpetual, irrevocable, royalty-free basis to BMJ Publishing Group Ltd ("BMJ") its licensees and where the relevant Journal is co-owned by BMJ to the co-owners of the Journal, to publish the Work in this journal and any other BMJ products and to exploit all rights, as set out in our licence.

The Submitting Author accepts and understands that any supply made under these terms is made by BMJ to the Submitting Author unless you are acting as an employee on behalf of your employer or a postgraduate student of an affiliated institution which is paying any applicable article publishing charge ("APC") for Open Access articles. Where the Submitting Author wishes to make the Work available on an Open Access basis (and intends to pay the relevant APC), the terms of reuse of such Open Access shall be governed by a Creative Commons licence – details of these licences and which Creative Commons licence will apply to this Work are set out in our licence referred to above.

Other than as permitted in any relevant BMJ Author's Self Archiving Policies, I confirm this Work has not been accepted for publication elsewhere, is not being considered for publication elsewhere and does not duplicate material already published. I confirm all authors consent to publication of this Work and authorise the granting of this licence.

For peer review only

Do childhood socioeconomic circumstances moderate the association between childhood cognitive ability and all-cause mortality across the life course? Prospective observational study of the 36-Day Sample of the Scottish Mental Survey 1947.

Matthew H Iveson*[1,2], Drew Altschul[1,2], and Ian J Deary[2,3]

[1]MRC Mental Health Data Pathfinder team, The University of Edinburgh, Edinburgh, UK; [2]The Centre for Cognitive Ageing and Cognitive Epidemiology, Department of Psychology, The University of Edinburgh, Edinburgh, UK; [3]Lothian Birth Cohorts, Department of Psychology, The University of Edinburgh, Edinburgh, UK.

Word count: 3706

Correspondence to:

Dr Matthew H. Iveson, Department of Psychology, The University of Edinburgh, 7 George Square, Edinburgh, EH8 9JZ, United Kingdom

Matthew.Iveson@ed.ac.uk

For peer review only

For peer review only - http://bmjopen.bmj.com/site/about/guidelines.xhtml

Abstract

Background

There is growing evidence that higher childhood cognitive ability predicts lower all-cause mortality risk across the life course. Whereas this association does not appear to be mediated by childhood socioeconomic circumstances, it is unclear whether socioeconomic circumstances moderate this association.

Methods

The moderating role of childhood socioeconomic circumstances was assessed in 5318 members of the 36-day sample of the Scottish Mental Survey 1947. Univariate, sex and age-adjusted, and mutually-adjusted Cox models predicting all-cause mortality risk up to age 79 were created using childhood IQ scores and childhood social class as predictors. Moderation was assessed by adding an interaction term between IQ scores and social class and comparing model fit.

Results

A standard deviation advantage in childhood IQ scores [Hazard Ratio (HR) = 0.83, 95% Confidence Interval (CI) = 0.79-0.86, $p < 0.001$] and a single class advantage in childhood social class [HR = 0.92, 95% CI = 0.88-0.97, $p < 0.001$] independently predicted lower mortality risk. Adding the IQ-social class interaction effect did not improve model fit [$X^2\Delta = 1.28$, $p = 0.26$], and the interaction effect did not predict mortality risk [HR = 1.03, 95% CI = 0.97-1.07, $p = 0.257$].

Conclusions

The present study demonstrated that the association between higher childhood cognitive ability and lower all-cause mortality risk is not conditional upon childhood social class. Whereas other measures of socioeconomic circumstances may play a moderating role, these findings suggest that the benefits of higher childhood cognitive ability for longevity apply regardless of the material socioeconomic circumstances experienced in childhood.

For peer review only - http://bmjopen.bmj.com/site/about/guidelines.xhtml

Keywords:

Mortality; Intelligence; Socioeconomic circumstances; Moderation effects

Article Summary

Strengths and limitations of this study

- All-cause mortality risk was examined in a large birth cohort over an entire life course.
- The study used contemporaneously-measured indicators of both childhood socioeconomic circumstances and childhood cognitive ability.
- The present study used a more fine-grained measure of childhood socioeconomic circumstances than previous research that better captures material resources in early life.
- Use of father's social class as a measure of childhood socioeconomic circumstances means potential miscoding of those whose father had a missing occupation.
- The particular social, economic and educational changes experienced by these older adults may limit the generalisability to younger cohorts.

For peer review only

Introduction

Several studies have demonstrated an association between higher cognitive ability and lower risk of all-cause mortality (1–3). This association stretches across the life-course, with childhood cognitive ability predicting mortality risk up to much later in life (4–8). There have been several attempts to model these associations while accounting for a suite of demographic and psychosocial factors as covariates. Perhaps being the most common covariate to be examined, some studies have reported that childhood socioeconomic circumstances mediate or confound the association between early-life cognitive ability and all-cause mortality risk either very slightly or not at all (6,7,9–11). However, the commonality between these studies has been the tendency to focus on examining only confounding and mediation effects while ignoring potential moderation of the cognitive ability-mortality association by socioeconomic circumstances. Whereas a mediator explains the association between two other variables (i.e., between childhood cognitive ability and all-cause mortality), a moderator influences the strength of the association between the variables. That is, the strength of the association between childhood cognitive ability and all-cause mortality risk may be conditional on socioeconomic circumstances; a strong association may be observed for some levels of socioeconomic circumstances, but a weaker (or no) association may be observed for others.

Only a few studies have considered the moderating role of socioeconomic circumstances in the association between cognitive ability and all-cause mortality. In a study using the Helsinki Birth Cohort, higher cognitive ability at age 20-years was associated with lower risk of mortality in later-life, and this association was stronger among those from middle-class than manual-occupational-class families (12). Similar moderation has been shown in the US National Longitudinal Survey of Youth 1979, albeit using parental education instead of occupational class (13). In that study, the association between higher cognitive ability in young adulthood and lower all-cause mortality risk was significant only for those whose parents had high school qualifications or above, and not for those whose parents had not completed high school. In these studies, better socioeconomic

For peer review only

circumstances is thought to allow access to the lifestyle and resources that enable higher cognitive abilities to impact upon health, such as through healthier diet and more physical activity (12). At the same time, lower socioeconomic circumstances may override much of the protective contribution of higher cognitive ability through exposure to other associated risk factors (13). However, these two studies are limited in terms of their samples – such as examining only males (12) – and in terms of their mortality follow-up – such as covering only mid-life mortality (13). Given the established sex differences in mortality risk (1,5,13), it is important to assess the moderation of the childhood cognitive ability-mortality risk association in a more population-representative sample. Furthermore, given that mortality risk increases with age and that childhood cognitive ability and socioeconomic circumstances both predict mortality risk in older adults (6,8), a longer follow-up is necessary to fully capture the contribution of a moderation effect. These studies are also limited by their use of early adulthood cognitive ability as a predictor of mortality, whereas many studies report a strong association between cognitive ability in childhood and mortality across the life course (4–7). Cognitive ability measured in childhood has the advantage of being up-stream of many of the factors that may confound the contribution of later cognitive ability, such as education (14). Although there appears to be little evidence that childhood socioeconomic circumstances mediate the association between childhood cognitive ability and all-cause mortality risk, their role as a moderator of this association remains unclear.

In the present study we examine the role of childhood socioeconomic circumstances as a possible moderator of the association between childhood cognitive ability and all-cause mortality risk across the life course. In particular, we utilise a representative sample of men and women born in Scotland in 1936 and whose cognitive ability was assessed as part of the Scottish Mental Survey 1947 and who were followed for mortality up to 2015. Importantly, an association between childhood cognitive ability and all-cause mortality has already been established in this sample (HR = 0.80, 95% CI [0.78, 0.81]; 6, 14) and in a subsample of these individuals (HR = 0.76, 95% CI [0.68, 0.84]; 7); however, potential moderation by childhood socioeconomic circumstances has not been

For peer review only - http://bmjopen.bmj.com/site/about/guidelines.xhtml

investigated. Given previous observations using adult cognitive ability (12,13), we predicted that childhood socioeconomic circumstances would significantly moderate the association between childhood cognitive ability and all-cause mortality, with stronger associations in those whose fathers had less manual-based occupations.

Methods

Sample

The sample consisted of 6291 individuals from the 36-day sample of the Scottish Mental Survey 1947 (16). These individuals, born on one of 36 birth dates in 1936 (the first three days of each month), took part in a nationwide test of cognitive ability on the 4th of June 1947 (age 11). They have been shown to be representative of the wider Scottish Mental Survey 1947 cohort in terms of childhood cognitive ability and socioeconomic conditions (16). The 36-day sample were subsequently followed-up across the life course, both in person and remotely using routinely-collected data (14,16,17). In particular, these individuals were traced in national migration and death records.

To create the analytic sample (N = 5318), we removed individuals with missing childhood cognitive ability (N = 575), missing childhood socioeconomic circumstances (N = 130), missing vitality status (N = 312) or missing date of death (if deceased, N = 12). Note that these individuals could have more than one key variable missing. Individuals who emigrated after completing the Scottish Mental Survey 1947 but before the end of the surveillance period were retained and censored appropriately. The study was approved by the local ethics and conformed to the Declaration of Helsinki.

Measures

Cognitive ability

For peer review only

Childhood cognitive ability around age 11 years was assessed using the Moray House Test No. 12 test of intelligence (MHT; 15) administered in groups as part of the Scottish Mental Survey 1947. The MHT consists of 71 items including: verbal reasoning, word classification, mixed sentences, same-opposites, analogies, proverbs, arithmetic, cypher decoding, following directions, proverbs, spatial problem-solving and practical items. Further details of the MHT and its administration are provided elsewhere (18). Raw MHT scores (ranging from 0 to 76) were converted to IQ-type score (Mean = 100, SD = 15) and then standardised (z-transformed).

Socioeconomic circumstances

The occupational social class of the father was used as a proxy of childhood socioeconomic circumstances at age 11 years. Father's occupation was recorded in 1947 as part of the Sociological Schedule – a planned follow-up to the Scottish Mental Survey 1947, completed mostly by head-teachers and school medical staff (16). Note that this was based on their current or most recent occupation. The most recent occupation of fathers who were without occupations, were unemployed, or were absent or deceased was recorded where possible; otherwise occupation was recorded as missing. Father's occupation was later categorised into one of 5 occupational social classes according to the 1950 United Kingdom's classification index (19). The social classes were reversed so that higher-numbered classes represented more professional occupations: 1) Unskilled, 2) Partly-skilled, 3) Skilled, 4) Managerial, 5) Professional.

Mortality

All-cause mortality (status and date of death) was obtained from National Records of Scotland death records covering 1947 to 2015. The linkage and extraction of death records have been described in detail elsewhere (7,17). Individuals were censored at the end of mortality surveillance (30thth

For peer review only - http://bmjopen.bmj.com/site/about/guidelines.xhtml

November 2015). Survival time was calculated as the number of days between 4th June 1947 and death or censoring date, as appropriate.

Analyses

We first described the demographic characteristics of the analytic sample, split by mortality status, before examining cohort selection effects by comparing excluded and retained individuals.

We then conducted survival analyses using Cox proportional hazards regression. All models incorporated inverse probability weights to adjust for sample selection effects (sex, age in days at Scottish Mental Survey 1947, z-transformed IQ scores and father's occupational social class as predictors of analytic sample membership; 36-Day Sample: weight mean = 1.98, SD = 4.14; Analytic sample: weight mean = 1.05, SD = 0.01). Hazard ratios were estimated to indicate the proportional change in all-cause mortality risk associated with a unit change in each predictor. As in previous work, individual models were constructed to assess the association between standardised IQ scores and reversed father's occupational social class (separately) and mortality risk. Consistent with previous work (13), models were then adjusted for sex to account for well-established sex differences in mortality risk and age in days at Scottish Mental Survey 1947 to account for small variations in age. A mutually-adjusted model was then created – including cognitive ability, social class and sex. Novel to this study, we further constructed a model additionally including an interaction term between cognitive ability and social class. In addition to examining predictors within a given model, the effect of adding a given predictor was assessed by comparing the fit of successive models (e.g., mutually-adjusted vs mutually-adjusted and moderated) using Chi-squared tests. Univariate survival models including standardised IQ score were then created for each social class in order to better assess interaction effects (or lack thereof).

For peer review only

For peer review only - http://bmjopen.bmj.com/site/about/guidelines.xhtml

Analysis was conducted in R (20) using RStudio (21), and with the 'psych' (22) and 'survival' (23) packages.

The analysis plan was preregistered prior to the data being analysed (https://osf.io/u8y9n/). The only change to the planned analysis was the treatment of father's occupational social class as numeric (not centred or transformed) during the survival analyses, rather than ordinal. This was done because use of the ordinal variable violated the proportional hazards assumption, and use of a numeric variable provided similar model fit to a categorical variable.

Patient and public involvement

This study was based on analysis of previously collected and anonymised research data and routinely-collected health data. As such, neither participants nor the public were involved in the design or planning of this study.

Results

Descriptive statistics for the analytic sample, split by father's occupational social class, are shown in Table 1. Mortality status ($p < 0.001$), survival times ($p < 0.001$) and MHT scores ($p < 0.001$) significantly differed between social classes, with a higher proportion of those in higher social classes being alive at censor date (Professional = 69.57%, Unskilled = 55.64%), surviving longer (Professional vs. Unskilled Mean difference = 3.74 years), and demonstrating higher MHT scores (Professional vs. Unskilled Mean difference = 16.97). Note, however, that the mean survival time of individuals with fathers from skilled occupations was lower than those with fathers from semi-skilled occupations, despite a lower proportion of deceased individuals. Furthermore, there were relatively few individuals with a father from a professional occupation. MHT scores were significantly and positively correlated with both father's occupational social class (Spearman's rho = 0.19, $p < 0.001$)

For peer review only - http://bmjopen.bmj.com/site/about/guidelines.xhtml

and survival time (Spearman's rho = 0.07, $p < 0.001$). Likewise, social class was significantly and positively correlated with survival time (Spearman's rho = 0.04, $p < 0.01$).

For peer review only

For peer review only - http://bmjopen.bmj.com/site/about/guidelines.xhtml

Table 1. Descriptive statistics for the analytic sample, split by father's occupational social class.

	Unskilled (N = 1073)	Semi-skilled (N = 1026)	Skilled (N = 2701)	Intermediate (N = 472)	Professional (N = 46)	Total (N = 5318)
**Sex**						
N (%) Male	546 (50.89%)	530 (51.66%)	1332 (49.32%)	239 (50.64%)	21 (45.65%)	2668 (50.17%)
N (%) Female	527 (49.11%)	496 (48.34%)	1369 (50.68%)	233 (49.36%)	25 (54.35%)	2650 (49.83%)
**Moray House Test of Intelligence Score (range = 0-74)** Mean (SD)	31.88 (15.39)	33.67 (15.57)	38.21 (15.18)	39.74 (15.91)	48.85 (12.69)	36.29 (15.65)
**Mortality status**						
N (%) Alive	597 (55.64%)	632 (61.60%)	1703 (63.05%)	321 (68.01%)	32 (69.57%)	3285 (61.77%)
N (%) Dead	476 (44.36%)	394 (38.40%)	998 (36.95%)	151 (31.99%)	14 (30.43%)	2033 (38.23%)
**Time from SMS1947 to death/censor (years)** Mean (SD)	57.14 (16.82)	57.78 (17.01)	56.79 (18.16)	59.96 (15.65)	60.88 (16.62)	57.37 (17.47)

SMS1947 = Scottish Mental Survey 1947

For peer review only

We then examined selection effects by comparing MHT scores and social class between the analytic sample and those removed due to having missing key variables. Note that this comparison necessarily included only those with at least one complete variable (e.g., a complete MHT score but missing father's social class). Removed individuals had significantly lower MHT score (removed mean = 34.42, $t(454) = -2.22$, $p < 0.05$), but the samples did not significantly differ in terms of social class ($X^2 = 9.30$, $p = 0.05$). This trend has been reported elsewhere (24,25) and may indicate that individuals with lower childhood cognitive ability were more likely to have long-term unemployed fathers who could not be ascribed an occupational social class. Note, however, that differences in MHT score were small; removed individuals scored on average 1.87 points fewer on the MHT.

Returning to the analytic sample, we next compared descriptive statistics between those alive (N = 3285) and those dead (N = 2033) (Table 1). In terms of sex, proportionately more males had died than females ($p < 0.001$). Those who died exhibited significantly lower MHT scores (M = 33.40, SD = 15.89) and tended to be from more manual social classes (Unskilled = 23.41%) than those who survived (MHT: M = 38.07, SD = 15.23, $p < 0.001$; Social class: Unskilled = 18.17%, $p < 0.001$).

We then conducted survival analyses aimed at examining whether social class moderated the association between childhood cognitive ability and all-cause mortality risk. Results from the Schoenfeld test and visual inspection of the scaled Schoenfeld residuals supported the proportional hazard assumption for all predictors in each model (once ordinal social class was replaced with numeric social class). Kaplan-Meier curves showing the association between IQ and survival within each social class are shown in Figure 1.

--Insert Figure 1 here--

For peer review only - http://bmjopen.bmj.com/site/about/guidelines.xhtml

Before examining moderation effects, we tested the independent associations of age-11 IQ and father's social class with all-cause mortality risk, as in previous work. In the univariate models, a 1SD advantage in age-11 IQ predicted a 19% lower mortality risk over time (Table 2). A 1-class advantage in father's social class predicted an 11% lower mortality risk over time. The strength of these associations was similar after adjusting for sex and age at Scottish Mental Survey 1947, though adding sex and age significantly improved the fit of each model (ps < 0.001). Likewise, mutual-adjustment (including sex and age) did not wholly or partly attenuate any one association with mortality risk, and significantly improved model fit versus both sex-adjusted models (ps < 0.001). A 1SD advantage in age-11 IQ and a 1-class advantage in father's social class were both independently associated with small-moderate reductions in all-cause mortality risk (18% and 7%, respectively; Table 2).

For peer review only - http://bmjopen.bmj.com/site/about/guidelines.xhtml

Table 2. Results from weighted Cox regression models predicting all-cause mortality risk. Regression coefficients and hazard ratios (HR) for IQ score and Father's social class in univariate models, models adjusted for sex and age at Scottish Mental Survey 1947 and models adjusted for both IQ score and Father's social class (and sex and age).

	Univariate[1,2]				Sex and age-adjusted[3,4]				Mutually-adjusted[5]			
**Predictor**	**B**	**SE**	**HR [95% CI]**	**p**	**B**	**SE**	**HR [95% CI]**	**p**	**B**	**SE**	**HR [95% CI]**	**p**
IQ score (1 SD)	-0.21	0.02	0.81 [0.77-0.84]	<0.001	-0.21	0.02	0.81 [0.78-0.85]	<0.001	-0.19	0.02	0.82 [0.79-0.86]	<0.001
Father's social class (1 class)	-0.12	0.02	0.89 [0.85-0.93]	<0.001	-0.12	0.02	0.89 [0.85-0.93]	<0.001	-0.08	0.02	0.93 [0.88-0.97]	<0.001

[1]Univariate IQ model R^2 = 0.02; [2]Univariate Father's social class model R^2 = 0.01; [3]Sex and age-adjusted IQ model R^2 = 0.04; [4]Sex and age-adjusted Father's social class model R^2 = 0.02; [5]Mutually-adjusted model R^2 = 0.04.

For peer review only

We then tested the moderating effect of father's social class. Adding the interaction term (age-11 IQ by father's social class) did not significantly improve model fit versus the mutually-adjusted model (Mutually-adjusted AIC = 35269.44, Interaction AIC = 35270.08; $X^2\Delta$ = 1.36, p = 0.24; Interaction R^2 = 0.04). The interaction effect did not significantly predict all-cause mortality risk, with 95% CIs that included 1 (B = 0.03, SE = 0.02, HR = 1.03, 95% CI [0.98-1.07], p = 0.25). Adding the interaction term further introduced a multicollinearity issue (VIF = 6.86) – strong collinearity between predictors (sex, father's social class, age-11 IQ and the interaction term) indicating some redundancy – which was not present in the mutually-adjusted model (all VIFs < 1.07).

Finally, we examined the association between age-11 IQ and all-cause mortality risk in each of the social classes (Table 3). A 1SD advantage in age-11 IQ significantly predicted moderately-reduced mortality risk among those from unskilled (22% decrease), semi-skilled (17% decrease) and skilled (17% decrease) backgrounds. The same direction of association, albeit not statistically significant and with wider 95% CIs that included 1, was observed among those from intermediate backgrounds. There was also no significant association between age-11 IQ and mortality risk among those from professional backgrounds, though this group was notably small (N = 46). Confidence intervals for this group were much wider than for other social classes. Although not part of the pre-registered analyses plan, at the advice of a reviewer we examined IQ-mortality associations among those removed individuals with a missing social class (but complete other variables) as these individuals may represent a specific at-risk group. There was a marginally-significant IQ-mortality association such that a 1SD advantage in IQ was associated with a 27% decrease in mortality risk. Note, however, that this group was particularly variable – with wide confidence intervals and a large standard error – reflecting its heterogeneity.

Table 3. Regression coefficients and hazard ratios (HR) for IQ score, adjusted for sex and age at Scottish Mental Survey 1947, in each of the Father's social classes.

	**B**	**SE**	**HR [95% CI]**	**p**	**R^2**	**AIC**
Unskilled (N = 1073)	-0.25	0.05	0.78 [0.71-0.85]	<0.001	0.05	6668.66
Semi-skilled (N = 1026)	-0.19	0.05	0.83 [0.75-0.91]	<0.001	0.03	5526.34

For peer review only - http://bmjopen.bmj.com/site/about/guidelines.xhtml

Skilled (N = 2701)	-0.19	0.03	0.83 [0.78-0.88]	<0.001	0.03	15797.24
Intermediate (N = 472)	-0.13	0.08	0.88 [0.75-1.03]	0.12	0.03	1873.17
Professional (N = 46)	0.13	0.35	1.13 [0.57-2.26]	0.72	0.05	112.63
Missing social class (N = 109)[1]	-0.32	0.17	0.73 [0.52, 1.01]	0.06	0.04	319.87

[1]Includes individuals with missing father's social class but with complete MHT scores and vitality status.

In further exploratory analyses suggested by reviewers we observed no significant 3-way interaction between IQ z-score, father's social class and sex (Supplementary Material). Furthermore, a consistent pattern of results was observed when re-analysing the data using additive regression models (Supplementary Material).

Discussion

The present study aimed to establish whether the association between higher childhood cognitive ability and reduced all-cause mortality risk is moderated by childhood socioeconomic circumstances. Whereas advantage in terms of childhood cognitive ability and father's social class independently predicted reduced risk of all-cause mortality, there was no evidence of a significant interaction effect in the whole-sample analysis. Although, in the sub-group analysis, we did observe a significant association between childhood cognitive ability-mortality risk in lower but not higher socioeconomic backgrounds we did not explicitly compare associations between classes. These results support previous observations that advantage in cognitive ability and socioeconomic circumstances have separate contributions to lower all-cause mortality risk (6,7), and further suggests that the benefits of high cognitive ability apply regardless of social class.

The lack of a significant moderation effect by socioeconomic circumstances is contrary to our hypothesis and the few previous studies to examine this (12,13). Notably, Jokela et al. (13) observed a significant (albeit small) moderating effect of parental education, with a stronger cognitive ability-mortality association among those with more educated parents, rather than occupational social class

For peer review only

For peer review only - http://bmjopen.bmj.com/site/about/guidelines.xhtml

as used here. Whereas parental education is thought to reflect the intellectual resources of the family, parental occupation is more indicative of the material resources of the family (26). The benefits of higher cognitive ability for health, therefore, may not depend on the material resources available in childhood. Whereas Kajantie et al. (12) did observe a significant moderation effect using parental occupational social class, only two categories were used (manual and middle-class). Such a coarse measure of occupational social class may overestimate its contribution to all-cause mortality risk by shifting the estimates for each category towards the mean of included classes – downwards for lower social classes and upwards for higher social classes. Furthermore, it assumes no differences between social classes within each category; here, the mortality risk associated with skilled, intermediate and professional occupational classes was very different.

The main strength of the present study is the use of both socioeconomic circumstances and cognitive ability measured contemporaneously in childhood. In the present study, advantage in these factors independently predicted reduced mortality risk, with no evidence of multicollinearity or confounding by sex. Although we only included one potential confounder, a more thorough investigation of confounding using other factors is available elsewhere (9–11), including in the same Scottish Mental Survey 1947 subsamples (6,7,14,15). Notably, childhood cognitive ability and childhood socioeconomic circumstances have been shown to predict later-life health, even after accounting for confounders such as education and socioeconomic circumstances in adulthood (5,6,25,27). Measuring these factors contemporaneously avoids potential recall bias associated with retrospective measures of socioeconomic circumstances in particular (28). Also, as individuals must survive until cognitive and socioeconomic measures are taken, examining these factors in childhood lessens potential selection effects (e.g., lower cognitive ability individuals who may die prior to adulthood). Furthermore, the present study examined all-cause mortality risk in a large sample over an entire life course (age 11-79 years-old). These individuals have had longer exposure to mortality risk than the younger samples used in previous work (12,13).

For peer review only - http://bmjopen.bmj.com/site/about/guidelines.xhtml

For peer review only

A limitation is that the sample is a product of the time and place in which they lived (29), and this may limit the generalisability of the results. All individuals in the sample were born in 1936 and lived in Scotland in 1947. They survived the Second World War and lived through the significant social, economic and educational changes that followed. Note that this also applies to previous work using the Helsinki Birth Cohort (born 1934-1944)(12). Childhood socioeconomic conditions (including occupational and educational opportunities of parents) in more modern samples, such as in the US National Longitudinal Survey of Youth(13), are likely better overall than those captured in the present sample (30). Indeed, relatively few individuals here originated from higher childhood social classes (intermediate or professional occupations). Furthermore, removal of individuals with missing childhood cognitive ability, childhood social class, or vitality status likely resulted in a sample slightly biased towards higher cognitive ability. Note also that the sample selection analyses does not include those with no complete key variables (i.e., all missing), who may constitute a very different population.

There are further limitations arising due to our use of father's occupational social class as a proxy measure of socioeconomic conditions. Firstly, although deceased, absent and long-term unemployed fathers were coded according to their most recent occupation where possible, the removal of individuals with a missing father's occupational social class likely results in a sample that does not represent the full range of socioeconomic conditions, though this is not a limitation that is unique to this study (12). Individuals raised in single parent households have been shown to be at higher risk of mortality (31), so removed individuals may represent a particularly vulnerable population. Secondly, while father's occupational social class is useful for this age of cohort it is perhaps less useful for younger cohorts for whom fathers are less likely to be the main breadwinners. More generally, the meaning of occupational social class is likely to have changed as new occupations are created and working conditions improved (32), thus the nature of the role of social class may be different in more modern social classes. Furthermore, these individuals had access to universal healthcare throughout their lives, unlike previous work using US-based samples (13), likely reducing the

For peer review only - http://bmjopen.bmj.com/site/about/guidelines.xhtml

For peer review only

importance of socioeconomic circumstances as both a predictor of mortality (33) and a moderator of the cognitive ability-mortality association. Finally, there are a number of unmeasured confounders, such as childhood health and earlier socioeconomic conditions, which may impact both childhood cognitive ability and mortality risk (9).

Consistent with previous work, the present study demonstrates an association between higher cognitive ability and lower all-cause mortality risk. However, it also suggests that this association is not conditional on one's social class of origin. Whereas childhood social class was an important independent predictor of all-cause mortality risk, there was no evidence that it moderated the role of childhood cognitive ability in predicting longevity. The benefits of higher childhood cognitive ability for lower all-cause mortality risk were observed across childhood social classes. This is an important consideration for public health policy and interventions: childhood cognitive ability is a source of health inequality independent from material socioeconomic resources in childhood, and needs to be addressed separately. This being said, it is unclear whether other measures of childhood socioeconomic circumstances, such as parental education, play a role in moderating the benefits of higher childhood cognitive ability. Future work should focus on clarifying the mechanisms by which childhood cognitive ability and childhood socioeconomic conditions affect mortality risk.

Contributors

All authors contributed to the conceptualization and design of this study. MHI contributed to data curation and formal analysis. MHI, DA and IJD contributed to investigation, methodology, project administration, validation, visualisation, writing – original draft preparation, and writing – review & editing. IJD contributed to funding acquisition and supervision.

Data sharing

For peer review only - http://bmjopen.bmj.com/site/about/guidelines.xhtml

Historical data from the Scottish Mental Survey 1947 is available upon request from the Department of Psychology, The University of Edinburgh. Access is contingent on local governance approvals. Routinely-collected records, including national death records, are available and linkable through NHS Information Services Division and the eData Research and Innovation Service (https://www.isdscotland.org/Products-and-Services/EDRIS/). Access is contingent on ethical and Public Benefit and Privacy Panel approvals.

Ethics statement

This submission is covered by the ethics application "Lifelong health and wellbeing of the 'Scotland in Miniature' cohort: The 6-Day Sample of the Scottish Mental Survey" (REC# 12/SS/0024; Scotland A Research Ethics Committee).

Funding statement

This work was supported by a Medical Research Council Mental Health Data Pathfinder award [MRC-MC_PC_17209], and by a UK cross council Lifelong Health and Wellbeing Initiative [MRCG1001401], for which IJD is the principal investigator. MHI, DA and IJD are members of The University of Edinburgh Centre for Cognitive Ageing and Cognitive Epidemiology, part of the cross council Lifelong Health and Wellbeing Initiative [MR/K026992/1].

For peer review only

For peer review only - http://bmjopen.bmj.com/site/about/guidelines.xhtml

References

1. Korten AE, Jorm AF, Jiao Z, Letenneur L, Jacomb PA, Henderson AS, et al. Health, cognitive, and psychosocial factors as predictors of mortality in an elderly community sample. J Epidemiol Community Health. 1999;53(2):83–8.

2. Pavlik VN, De Moraes SA, Szklo M, Knopman DS, Mosley TH, Hymans DJ. Relation between cognitive function and mortality in middle-aged adults: The atherosclerosis risk in communities study. Am J Epidemiol. 2003;157(4):327–34.

3. Batterham PJ, Christensen H, Mackinnon AJ. Fluid intelligence is independently associated with all-cause mortality over 17 years in an elderly community sample: An investigation of potential mechanisms. Intelligence [Internet]. 2009;37(6):551–60. Available from: http://dx.doi.org/10.1016/j.intell.2008.10.004

4. Bratsberg B, Rogeberg O. Childhood socioeconomic status does not explain the IQ-mortality gradient. Intelligence [Internet]. 2017;62(March):148–54. Available from: http://dx.doi.org/10.1016/j.intell.2017.04.002

5. Calvin CM, Deary IJ, Fenton C, Roberts BA, Der G, Leckenby N, et al. Intelligence in youth and all-cause-mortality: Systematic review with meta-analysis. Int J Epidemiol. 2011;40(3):626–44.

6. Calvin CM, Batty GD, Der G, Brett CE, Taylor A, Pattie A, et al. Childhood intelligence in relation to major causes of death in 68 year follow-up: Prospective population study. BMJ. 2017;357.

7. Iveson MH, Čukič I, Der G, Batty GD, Deary IJ. Intelligence and all-cause mortality in the 6-Day Sample of the Scottish Mental Survey 1947 and their siblings: Testing the contribution of family background. Int J Epidemiol. 2018;47(1).

8. Whalley LJ, Deary IJ. Longitudinal cohort study of childhood IQ and survival up to age 76. Br

For peer review only - http://bmjopen.bmj.com/site/about/guidelines.xhtml

Med J. 2001;322(7290):819–22.

9. Kuh D, Richards M, Hardy R, Butterworth S, Wadsworth MEJ. Childhood cognitive ability and deaths up until middle age: A post-war birth cohort study. Int J Epidemiol. 2004;33(2):408–13.

10. Batty GD, Wennerstad KM, Smith GD, Gunnell D, Deary IJ, Tynelius P, et al. IQ in early adulthood and mortality by middle age: Cohort study of 1 million swedish men. Epidemiology. 2009;20(1):100–9.

11. Lager A, Bremberg S, Vågerö D. The association of early IQ and education with mortality: 65 Year longitudinal study in Malmö, Sweden. BMJ. 2009;339(7735):1432.

12. Kajantie E, Räikkönen K, Henriksson M, Forsén T, Heinonen K, Pesonen AK, et al. Childhood socioeconomic status modifies the association between intellectual abilities at age 20 and mortality in later life. J Epidemiol Community Health. 2010;64(11):963–9.

13. Jokela M, Elovainio M, Singh-Manoux A, Kivimäki M. IQ, Socioeconomic status, and early death: The US national longitudinal survey of youth. Psychosom Med. 2009;71(3):322–8.

14. Deary IJ, Brett CE. Predicting and retrodicting intelligence between childhood and old age in the 6-Day Sample of the Scottish Mental Survey 1947. Intelligence [Internet]. 2015;50:1–9. Available from: http://dx.doi.org/10.1016/j.intell.2015.02.002

15. Čukić I, Brett CE, Calvin CM, Batty GD, Deary IJ. Childhood IQ and survival to 79: Follow-up of 94% of the Scottish Mental Survey 1947. Intelligence. 2017;63(May):45–50.

16. The Scottish Council for Research in Education. The trend of Scottish intelligence. London: University of London Press; 1949.

17. Brett CE, Deary IJ. Realising health data linkage from a researcher’s perspective: following up the 6-Day Sample of the Scottish Mental Survey 1947. Longit Life Course Stud. 2014;5(3):283–

For peer review only - http://bmjopen.bmj.com/site/about/guidelines.xhtml

98.

18. Deary IJ, Whiteman MC, Starr JM, Whalley LJ, Fox HC. The Impact of Childhood Intelligence on Later Life: Following Up the Scottish Mental Surveys of 1932 and 1947. J Pers Soc Psychol. 2004;86(1):130–47.

19. General Register Office. Census, 1951: Classification of Occupations. London: HMSO; 1956.

20. R Core Team. R: A language and environment for statistical computing. Vienna: R Foundation for Statistical Computing; 2018.

21. RStudio Team. RStudio: Integrated development for R. Boston, MA: RStudio Inc.; 2016.

22. Revelle W. psych: Procedures for personality and psychological research. Evanston: Northwestern University; 2018.

23. Therneau TM, Lumley T. Package “survival.” R Top Doc. 2015;128.

24. Johnson W, Brett CE, Calvin C, Deary IJ. Childhood characteristics and participation in Scottish Mental Survey 1947 6-Day Sample Follow-ups: Implications for participation in aging studies. Intelligence [Internet]. 2016;54:70–9. Available from: http://dx.doi.org/10.1016/j.intell.2015.11.006

25. Iveson MH, Deary IJ. Intergenerational social mobility and subjective wellbeing in later life. Soc Sci Med [Internet]. 2017;188:11–20. Available from: http://dx.doi.org/10.1016/j.socscimed.2017.06.038

26. Galobardes B, Shaw M, Lawlor DA, Lynch JW, Smith GD. Indicators of socioeconomic position (part 1). J Epidemiol Community Health. 2006;60(1):7–12.

27. Iveson MH, Deary IJ. Early-life predictors of retirement decisions and post-retirement health. SSM - Popul Heal [Internet]. 2019;8(February):100430. Available from: https://doi.org/10.1016/j.ssmph.2019.100430

For peer review only

For peer review only - http://bmjopen.bmj.com/site/about/guidelines.xhtml

28. Batty GD, Lawlor DA, Macintyre S, Clark H, Leon DA. Accuracy of adults' recall of childhood social class: Findings from the Aberdeen children of the 1950s study. J Epidemiol Community Health. 2005;59(10):898–903.

29. Elder Jr GH. Time, human agency, and social change: Perspectives on the life course. Soc Psychol Q. 1994;4–15.

30. Galobardes B, Lynch JW, Smith GD. Childhood socioeconomic circumstances and cause-specific mortality in adulthood: Systematic review and interpretation. Epidemiol Rev. 2004;26:7–21.

31. Weitoft GR, Hjern A, Haglund B, Rosén M. Mortality, severe morbidity, and injury in children living with single parents in Sweden: A population-based study. Lancet. 2003;361(9354):289–95.

32. Goldthorpe JH. On Economic Development and Social Mobility. Br J Soc. 1985;36(4):549–73.

33. Asaria M, Ali S, Doran T, Ferguson B, Fleetcroft R, Goddard M, et al. How a universal health system reduces inequalities: Lessons from England. J Epidemiol Community Health. 2016;70(7):637–43.

For peer review only

Figure 1. Kaplan-Meier Survival curves showing survival probability associated with IQ z-scores 1 SD below (red), around (green) or above (blue) the mean, split by father's occupational social class.

For peer review only

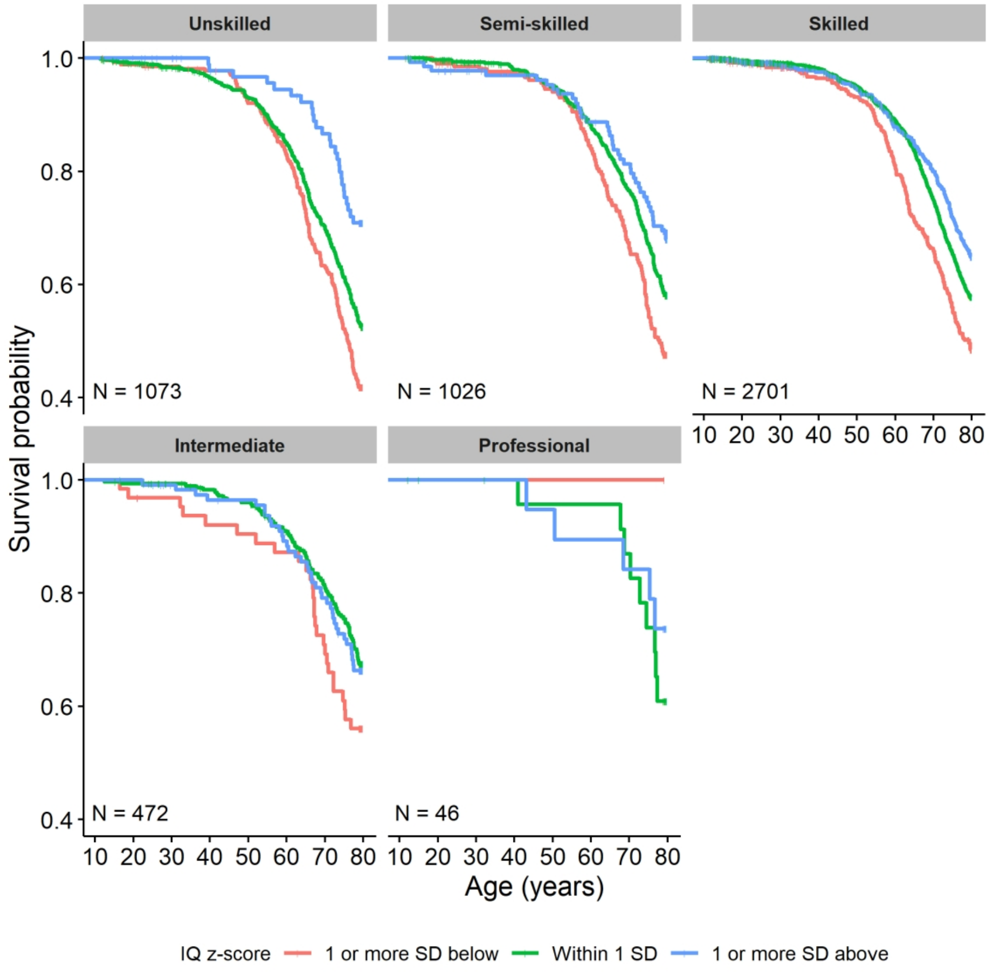

Figure 1. Kaplan-Meier Survival curves showing survival probability associated with IQ z-scores 1 SD below (red), around (green) or above (blue) the mean, split by father's occupational social class.

179x179mm (300 x 300 DPI)

Supplementary Material

3-way Interaction

At the request of a reviewer, we additionally performed an exploratory analysis of a three-way interaction between IQ z-scores, father's occupational social class (reversed, continuous) and sex. In particular, the aim was to test whether the moderating role of childhood socioeconomic circumstances itself depended on sex.

This was done by constructing a Cox regression model that included main effects of IQ z-scores, father's social class (reversed, continuous), sex and age in days at the Scottish Mental Survey 1947, two-way interactions between IQ z-scores X father's social class, IQ z-scores X sex and father's social class X sex, as well as the three-way interaction between IQ z-scores X father's social class X sex.

Including these additional interaction effects resulted in multicollinearity issues (all interaction VIFs > 8.50). Furthermore, it did not significantly improve model fit versus the IQ z-score X father's social class model included in the main text (AIC = 35275.82; $X^2\Delta = 0.26$, $p = 0.97$; Interaction $R^2 = 0.04$). None of the included interactions significantly predicted all-cause mortality risk (Table A1).

Table A1. Results from weighted Cox regression model, adjusted for age in days at Scottish Mental Survey 1947, predicting all-cause mortality risk. Regression coefficients and hazard ratios (HR) for IQ score, Father's social class, Sex, IQ X Father's social class, IQ X Sex, Father's social class X Sex, IQ X Father's social class X Sex.

**Predictor**	**B**	**SE**	**HR [95% CI]**	**p**
IQ score (1 SD)	-0.26	0.07	0.77 [0.67-0.89]	<0.001
Father's social class (1 class)	-0.07	0.03	0.94 [0.88-1.00]	0.04
Sex (Female)	-0.36	0.13	0.70 [0.54-0.90]	<0.01
IQ X Father's social class	0.02	0.03	1.03 [0.97-1.08]	0.38
IQ X Sex	0.01	0.12	1.01 [0.80-1.27]	0.94
Father's social class X Sex	-0.02	0.05	0.98 [0.89-1.08]	0.70
IQ X Father's social class X Sex	<0.01	0.04	1.00 [0.92-1.10]	0.94

Additive hazards model

At the request of a reviewer, we performed Additive Hazards regression using Aalen's additive regression models equivalent to the Cox Proportional Hazards models used in the main text. Additive models have the advantage of allowing the effects of covariates – including interaction effects – to vary over time (1). For example, advantage in childhood cognitive ability may benefit survival in particular socioeconomic groups but only in specific periods of

For peer review only - http://bmjopen.bmj.com/site/about/guidelines.xhtml

life. Additive models help to provide information about the effect in the context of the underlying hazard – a small hazard ratio for the interaction may still be important if the underlying hazard is large (1). We estimated the number of additional deaths per 10,000 person years predicted by each covariate of interest – IQ z-scores, father's occupational social class and the IQ-social class interaction. As in the main text, univariate, sex and age-adjusted, mutually-adjusted, and interaction models were constructed.

As with mortality risk in the main text, a 1SD advantage in IQ and a 1 class advantage in father's social class were both significantly associated with a small reduction in the number of deaths, even when adjusted for sex and age at time of the Scottish Mental Survey 1947 (Table A2). These associations remained significant when mutually-adjusting, supporting the suggestion that childhood cognitive ability and childhood socioeconomic circumstances independently predict mortality risk. In the final model, the IQ-social class interaction predicted a small increase in the number of deaths per 10,000 person years, though this was only marginally-significant (Table A2).

Additive models were constructed to examine the IQ-mortality association within each social class, including those whose father's occupational social class was missing, as in the main text (Table A3). The direction and pattern of associations was consistent with the Cox Proportional Hazards models used in the main text: a 1SD advantage in IQ was significantly associated with fewer deaths per person year among those from unskilled, semi-skilled and skilled social class backgrounds. The benefit of advantage in IQ appeared to diminish slightly as class increased, suggesting that high IQ may benefit those from lower social classes most. Note again, however, that the interaction effect in the whole-sample model above was not significant. There was no significant association between IQ and mortality in those from intermediate, professional or missing occupational social classes, and confidence intervals in these groups were wide.

We conclude that the additive hazards approach presented here is in keeping with the proportional hazards approach used in the main text.

For peer review only

Table A2. Results from weighted Additive Hazards regression models predicting all-cause mortality risk. Additional deaths per 10,000 person years and 95% Confidence Intervals for IQ score and Father's social class in univariate models, models adjusted for sex and age at Scottish Mental Survey 1947, models adjusted for both IQ score and Father's social class (and sex and age), and a model additionally including the IQ-Social class interaction effect (and age and sex).

	Univariate		Sex and age-adjusted		Mutually-adjusted		Interaction model	
**Predictor**	**Additional deaths per 10,000 person years [95% CI]**	**p**	**Additional deaths per 10,000 person years [95% CI]**	**p**	**Additional deaths per 10,000 person years [95% CI]**	**p**	**Additional deaths per 10,000 person years [95% CI]**	**p**
IQ score (1 SD)	-0.57 [-0.69, -0.45]	<0.001	-0.56 [-0.68, -0.44]	<0.001	-0.53 [-0.66, -0.41]	<0.001	-0.85 [-1.19, -0.50]	<0.001
Father's social class (1 class)	-0.31 [-0.43, -0.19]	<0.001	-0.31 [-0.43, -0.19]	<0.001	-0.20 [-0.33, -0.08]	<0.01	-0.21 [-0.33, -0.08]	<0.01
IQ X Father's social class	-	-	-	-	-	-	0.13 [<0.01, 0.25]	0.05

Table A3. Additional deaths per 10,000 person years and 95% Confidence Intervals for IQ score, adjusted for sex and age at Scottish Mental Survey 1947, in each of the Father's social classes.

	**Additional deaths per 10,000 person years [95% CI]**	**p**
Unskilled (N = 1073)	-3.37 [-4.58, -2.15]	<0.001
Semi-skilled (N = 1026)	-2.67 [-4.09, -1.24]	<0.001
Skilled (N = 2701)	-1.05 [-1.41, -0.68]	<0.001
Intermediate (N = 472)	-3.62 [-8.26, 1.02]	0.13
Professional (N = 46)	31.16 [-146.18, 208.50]	0.73
Missing social class (N = 109)[1]	-42.56 [-88.32, 3.19]	0.07

[1]Includes individuals with missing father's social class but with complete MHT scores and vitality status.

For peer review only

References

1. Rod NJ, Lange T, Andersen I, Marott JL, Diderichsen F. Additive interaction in survival analysis: Use of the additive hazards model. Epidemiology. 2012;23(5):733-737.

For peer review only

STROBE Statement—Checklist of items that should be included in reports of ***cohort studies***

	Item No	Recommendation	Page (Line)
**Title and abstract**	1	(*a*) Indicate the study's design with a commonly used term in the title or the abstract	1 (1)
		(*b*) Provide in the abstract an informative and balanced summary of what was done and what was found	2 (1)
**Introduction**			
Background/rationale	2	Explain the scientific background and rationale for the investigation being reported	4 (1)
Objectives	3	State specific objectives, including any prespecified hypotheses	5 (19)
**Methods**			
Study design	4	Present key elements of study design early in the paper	6 (7)
Setting	5	Describe the setting, locations, and relevant dates, including periods of recruitment, exposure, follow-up, and data collection	6 (7)
Participants	6	(*a*) Give the eligibility criteria, and the sources and methods of selection of participants. Describe methods of follow-up	6 (7)
		(*b*) For matched studies, give matching criteria and number of exposed and unexposed	NA
Variables	7	Clearly define all outcomes, exposures, predictors, potential confounders, and effect modifiers. Give diagnostic criteria, if applicable	6 (23)
Data sources/ measurement	8*	For each variable of interest, give sources of data and details of methods of assessment (measurement). Describe comparability of assessment methods if there is more than one group	6 (23)
Bias	9	Describe any efforts to address potential sources of bias	8 (7)
Study size	10	Explain how the study size was arrived at	6 (15)
Quantitative variables	11	Explain how quantitative variables were handled in the analyses. If applicable, describe which groupings were chosen and why	8 (7)
Statistical methods	12	(*a*) Describe all statistical methods, including those used to control for confounding	8 (4)
		(*b*) Describe any methods used to examine subgroups and interactions	8 (17)
		(*c*) Explain how missing data were addressed	6 (15)
		(*d*) If applicable, explain how loss to follow-up was addressed	7 (21)
		(*e*) Describe any sensitivity analyses	NA
**Results**			
Participants	13*	(a) Report numbers of individuals at each stage of study—eg numbers potentially eligible, examined for eligibility, confirmed eligible, included in the study, completing follow-up, and analysed	6 (7)
		(b) Give reasons for non-participation at each stage	6 (15)
		(c) Consider use of a flow diagram	NA
Descriptive data	14*	(a) Give characteristics of study participants (eg demographic, clinical, social) and information on exposures and potential confounders	9 (13)
		(b) Indicate number of participants with missing data for each variable of interest	6 (15)
		(c) Summarise follow-up time (eg, average and total amount)	7 (24)
Outcome data	15*	Report numbers of outcome events or summary measures over time	10
Main results	16	(*a*) Give unadjusted estimates and, if applicable, confounder-adjusted estimates and their precision (eg, 95% confidence interval). Make clear	13

For peer review only - http://bmjopen.bmj.com/site/about/guidelines.xhtml

		which confounders were adjusted for and why they were included	
		(*b*) Report category boundaries when continuous variables were categorized	NA
		(*c*) If relevant, consider translating estimates of relative risk into absolute risk for a meaningful time period	NA
Other analyses	17	Report other analyses done—eg analyses of subgroups and interactions, and sensitivity analyses	14 (1)
**Discussion**			
Key results	18	Summarise key results with reference to study objectives	15 (10)
Limitations	19	Discuss limitations of the study, taking into account sources of potential bias or imprecision. Discuss both direction and magnitude of any potential bias	17 (1)
Interpretation	20	Give a cautious overall interpretation of results considering objectives, limitations, multiplicity of analyses, results from similar studies, and other relevant evidence	18 (5)
Generalisability	21	Discuss the generalisability (external validity) of the study results	17 (1)
**Other information**			
Funding	22	Give the source of funding and the role of the funders for the present study and, if applicable, for the original study on which the present article is based	19 (13)

*Give information separately for exposed and unexposed groups.

Note: An Explanation and Elaboration article discusses each checklist item and gives methodological background and published examples of transparent reporting. The STROBE checklist is best used in conjunction with this article (freely available on the Web sites of PLoS Medicine at http://www.plosmedicine.org/, Annals of Internal Medicine at http://www.annals.org/, and Epidemiology at http://www.epidem.com/). Information on the STROBE Initiative is available at http://www.strobe-statement.org.

For peer review only

For peer review only - http://bmjopen.bmj.com/site/about/guidelines.xhtml

Do childhood socioeconomic circumstances moderate the association between childhood cognitive ability and all-cause mortality across the life course? Prospective observational study of the 36-Day Sample of the Scottish Mental Survey 1947.

Journal:	*BMJ Open*
Manuscript ID	bmjopen-2020-037847.R2
Article Type:	Original research
Date Submitted by the Author:	02-Nov-2020

Complete List of Authors:	Iveson, Matthew; The University of Edinburgh, Centre for Clinical Brain Sciences<br>Altschul, Drew; The University of Edinburgh, Department of Psychology<br>Deary, Ian; The University of Edinburgh, Department of Psychology

**Primary Subject Heading**:	Epidemiology
Secondary Subject Heading:	Epidemiology, Public health
Keywords:	EPIDEMIOLOGY, PUBLIC HEALTH, STATISTICS & RESEARCH METHODS

SCHOLARONE™
Manuscripts

I, the Submitting Author has the right to grant and does grant on behalf of all authors of the Work (as defined in the below author licence), an exclusive licence and/or a non-exclusive licence for contributions from authors who are: i) UK Crown employees; ii) where BMJ has agreed a CC-BY licence shall apply, and/or iii) in accordance with the terms applicable for US Federal Government officers or employees acting as part of their official duties; on a worldwide, perpetual, irrevocable, royalty-free basis to BMJ Publishing Group Ltd ("BMJ") its licensees and where the relevant Journal is co-owned by BMJ to the co-owners of the Journal, to publish the Work in this journal and any other BMJ products and to exploit all rights, as set out in our licence.

The Submitting Author accepts and understands that any supply made under these terms is made by BMJ to the Submitting Author unless you are acting as an employee on behalf of your employer or a postgraduate student of an affiliated institution which is paying any applicable article publishing charge ("APC") for Open Access articles. Where the Submitting Author wishes to make the Work available on an Open Access basis (and intends to pay the relevant APC), the terms of reuse of such Open Access shall be governed by a Creative Commons licence – details of these licences and which Creative Commons licence will apply to this Work are set out in our licence referred to above.

Other than as permitted in any relevant BMJ Author's Self Archiving Policies, I confirm this Work has not been accepted for publication elsewhere, is not being considered for publication elsewhere and does not duplicate material already published. I confirm all authors consent to publication of this Work and authorise the granting of this licence.

For peer review only

Do childhood socioeconomic circumstances moderate the association between childhood cognitive ability and all-cause mortality across the life course? Prospective observational study of the 36-Day Sample of the Scottish Mental Survey 1947.

Matthew H Iveson*[1,2], Drew Altschul[1,2], and Ian J Deary[2,3]

[1]MRC Mental Health Data Pathfinder team, The University of Edinburgh, Edinburgh, UK; [2]The Centre for Cognitive Ageing and Cognitive Epidemiology, Department of Psychology, The University of Edinburgh, Edinburgh, UK; [3]Lothian Birth Cohorts, Department of Psychology, The University of Edinburgh, Edinburgh, UK.

Word count: 3939

Correspondence to:

Dr Matthew H. Iveson, Department of Psychology, The University of Edinburgh, 7 George Square, Edinburgh, EH8 9JZ, United Kingdom

Matthew.Iveson@ed.ac.uk

For peer review only

For peer review only - http://bmjopen.bmj.com/site/about/guidelines.xhtml

Abstract

Background

There is growing evidence that higher childhood cognitive ability predicts lower all-cause mortality risk across the life course. Whereas this association does not appear to be mediated by childhood socioeconomic circumstances, it is unclear whether socioeconomic circumstances moderate this association.

Methods

The moderating role of childhood socioeconomic circumstances was assessed in 5318 members of the 36-day sample of the Scottish Mental Survey 1947. Univariate, sex and age-adjusted, and mutually-adjusted Cox models predicting all-cause mortality risk up to age 79 were created using childhood IQ scores and childhood social class as predictors. Moderation was assessed by adding an interaction term between IQ scores and social class and comparing model fit.

Results

A standard deviation advantage in childhood IQ scores [Hazard Ratio (HR) = 0.83, 95% Confidence Interval (CI) = 0.79-0.86, $p < 0.001$] and a single class advantage in childhood social class [HR = 0.92, 95% CI = 0.88-0.97, $p < 0.001$] independently predicted lower mortality risk. Adding the IQ-social class interaction effect did not improve model fit [$X^2\Delta = 1.36$, $p = 0.24$], and the interaction effect did not predict mortality risk [HR = 1.03, 95% CI = 0.98-1.07, $p = 0.25$].

Conclusions

The present study demonstrated that the association between higher childhood cognitive ability and lower all-cause mortality risk is not conditional upon childhood social class. Whereas other measures of socioeconomic circumstances may play a moderating role, these findings suggest that the benefits of higher childhood cognitive ability for longevity apply regardless of the material socioeconomic circumstances experienced in childhood.

For peer review only - http://bmjopen.bmj.com/site/about/guidelines.xhtml
For peer review only

Keywords:

Mortality; Intelligence; Socioeconomic circumstances; Moderation effects

Article Summary

Strengths and limitations of this study

- All-cause mortality risk was examined in a large birth cohort over an entire life course.
- The study used contemporaneously-measured indicators of both childhood socioeconomic circumstances and childhood cognitive ability.
- The present study used a more fine-grained measure of childhood socioeconomic circumstances than previous research that better captures material resources in early life.
- Use of father's social class as a measure of childhood socioeconomic circumstances means potential miscoding of those whose father had a missing occupation.
- The particular social, economic and educational changes experienced by these older adults may limit the generalisability to younger cohorts.

For peer review only

Introduction

Several studies have demonstrated an association between higher cognitive ability and lower risk of all-cause mortality (1–3). This association stretches across the life-course, with childhood cognitive ability predicting mortality risk up to much later in life (4–8). There have been several attempts to model these associations while accounting for a suite of demographic and psychosocial factors as covariates. Perhaps being the most common covariate to be examined, some studies have reported that childhood socioeconomic circumstances mediate or confound the association between early-life cognitive ability and all-cause mortality risk either very slightly or not at all (6,7,9–11). However, the commonality between these studies has been the tendency to focus on examining only confounding and mediation effects while ignoring potential moderation of the cognitive ability-mortality association by socioeconomic circumstances. Whereas a mediator explains the association between two other variables (i.e., between childhood cognitive ability and all-cause mortality), a moderator influences the strength of the association between the variables. That is, the strength of the association between childhood cognitive ability and all-cause mortality risk may be conditional on socioeconomic circumstances; a strong association may be observed for some levels of socioeconomic circumstances, but a weaker (or no) association may be observed for others.

Only a few studies have considered the moderating role of socioeconomic circumstances in the association between cognitive ability and all-cause mortality. In a study using the Helsinki Birth Cohort, higher cognitive ability at age 20-years was associated with lower risk of mortality in later-life, and this association was stronger among those from middle-class than manual-occupational-class families (12). Similar moderation has been shown in the US National Longitudinal Survey of Youth 1979, albeit using parental education instead of occupational class (13). In that study, the association between higher cognitive ability in young adulthood and lower all-cause mortality risk was significant only for those whose parents had high school qualifications or above, and not for those whose parents had not completed high school. In these studies, better socioeconomic

For peer review only - http://bmjopen.bmj.com/site/about/guidelines.xhtml

circumstances is thought to allow access to the lifestyle and resources that enable higher cognitive abilities to impact upon health, such as through healthier diet and more physical activity (12). At the same time, lower socioeconomic circumstances may override much of the protective contribution of higher cognitive ability through exposure to other associated risk factors (13). However, these two studies are limited in terms of their samples – such as examining only males (12) – and in terms of their mortality follow-up – such as covering only mid-life mortality (13). Given the established sex differences in mortality risk (1,5,13), it is important to assess the moderation of the childhood cognitive ability-mortality risk association in a more population-representative sample. Furthermore, given that mortality risk increases with age and that childhood cognitive ability and socioeconomic circumstances both predict mortality risk in older adults (6,8), a longer follow-up is necessary to fully capture the contribution of a moderation effect. These studies are also limited by their use of early adulthood cognitive ability as a predictor of mortality, whereas many studies report a strong association between cognitive ability in childhood and mortality across the life course (4–7). Cognitive ability measured in childhood has the advantage of being up-stream of many of the factors that may confound the contribution of later cognitive ability, such as education (14). Although there appears to be little evidence that childhood socioeconomic circumstances mediate the association between childhood cognitive ability and all-cause mortality risk, their role as a moderator of this association remains unclear.

In the present study we examine the role of childhood socioeconomic circumstances as a possible moderator of the association between childhood cognitive ability and all-cause mortality risk across the life course. In particular, we utilise a representative sample of men and women born in Scotland in 1936 and whose cognitive ability was assessed as part of the Scottish Mental Survey 1947 and who were followed for mortality up to 2015. Importantly, an association between childhood cognitive ability and all-cause mortality has already been established in this sample (HR = 0.80, 95% CI [0.78, 0.81]; 6,14,15) and in a subsample of these individuals (HR = 0.76, 95% CI [0.68, 0.84]; 7); however, potential moderation by childhood socioeconomic circumstances has not been

For peer review only - http://bmjopen.bmj.com/site/about/guidelines.xhtml

investigated. Given previous observations using adult cognitive ability (12,13), we predicted that childhood socioeconomic circumstances would significantly moderate the association between childhood cognitive ability and all-cause mortality, with stronger associations in those whose fathers had less manual-based occupations.

Methods

Sample

The sample consisted of 6291 individuals from the 36-day sample of the Scottish Mental Survey 1947 (16). These individuals, born on one of 36 birth dates in 1936 (the first three days of each month), took part in a nationwide test of cognitive ability on the 4th of June 1947 (age 11). They have been shown to be representative of the wider Scottish Mental Survey 1947 cohort in terms of childhood cognitive ability and socioeconomic conditions (15,16). The 36-day sample were subsequently followed-up across the life course, both in person and remotely using routinely-collected data (14,16,17). In particular, these individuals were traced in national migration and death records.

To create the analytic sample (N = 5318), we removed individuals with missing childhood cognitive ability (N = 575), missing childhood socioeconomic circumstances (N = 130), missing vitality status (N = 312) or missing date of death (if deceased, N = 12). Note that these individuals could have more than one key variable missing. Individuals who emigrated after completing the Scottish Mental Survey 1947 but before the end of the surveillance period were retained and censored appropriately. The study was approved by the local ethics and conformed to the Declaration of Helsinki.

Measures

Cognitive ability

For peer review only - http://bmjopen.bmj.com/site/about/guidelines.xhtml

Childhood cognitive ability around age 11 years was assessed using the Moray House Test No. 12 test of intelligence (MHT; 15) administered in groups as part of the Scottish Mental Survey 1947. The MHT consists of 71 items including: verbal reasoning, word classification, mixed sentences, same-opposites, analogies, proverbs, arithmetic, cypher decoding, following directions, proverbs, spatial problem-solving and practical items. Further details of the MHT and its administration are provided elsewhere (18). Raw MHT scores (ranging from 0 to 76) were converted to IQ-type score (Mean = 100, SD = 15) and then standardised (z-transformed).

Socioeconomic circumstances

The occupational social class of the father was used as a proxy of childhood socioeconomic circumstances at age 11 years. Father's occupation was recorded in 1947 as part of the Sociological Schedule – a planned follow-up to the Scottish Mental Survey 1947, completed mostly by head-teachers and school medical staff (16). Note that this was based on their current or most recent occupation. The most recent occupation of fathers who were without occupations, were unemployed, or were absent or deceased was recorded where possible; otherwise occupation was recorded as missing. Father's occupation was later categorised into one of 5 occupational social classes according to the 1950 United Kingdom's classification index (19). The social classes were reversed so that higher-numbered classes represented more professional occupations: 1) Unskilled, 2) Partly-skilled, 3) Skilled, 4) Managerial, 5) Professional.

Mortality

All-cause mortality (status and date of death) was obtained from National Records of Scotland death records covering 1947 to 2015. The linkage and extraction of death records have been described in detail elsewhere (7,17). Individuals were censored at the end of mortality surveillance (30thth

For peer review only - http://bmjopen.bmj.com/site/about/guidelines.xhtml

November 2015). Survival time was calculated as the number of days between 4th June 1947 and death or censoring date, as appropriate.

Analyses

We first described the demographic characteristics of the analytic sample, split by mortality status, before examining cohort selection effects by comparing excluded and retained individuals.

We then conducted survival analyses using Cox proportional hazards regression. All models incorporated inverse probability weights to adjust for sample selection effects (sex, age in days at Scottish Mental Survey 1947, z-transformed IQ scores and father's occupational social class as predictors of analytic sample membership; 36-Day Sample: weight mean = 1.98, SD = 4.14; Analytic sample: weight mean = 1.05, SD = 0.01). Hazard ratios were estimated to indicate the proportional change in all-cause mortality risk associated with a unit change in each predictor. As in previous work, individual models were constructed to assess the association between standardised IQ scores and reversed father's occupational social class (separately) and mortality risk. Consistent with previous work (13), models were then adjusted for two potential confounders: sex to account for well-established sex differences in mortality risk and age in days at Scottish Mental Survey 1947 to account for small variations in age. A mutually-adjusted model was then created – including cognitive ability, social class and sex. Novel to this study, we further constructed a model additionally including an interaction term between cognitive ability and social class. In addition to examining predictors within a given model, the effect of adding a given predictor was assessed by comparing the fit of successive models (e.g., mutually-adjusted vs mutually-adjusted and moderated) using Chi-squared tests. Univariate survival models including standardised IQ score were then created for each social class in order to better assess interaction effects (or lack thereof).

For peer review only

For peer review only - http://bmjopen.bmj.com/site/about/guidelines.xhtml

Analysis was conducted in R (20) using RStudio (21), and with the 'psych' (22) and 'survival' (23) packages.

The analysis plan was preregistered prior to the data being analysed (https://osf.io/u8y9n/). The only change to the planned analysis was the treatment of father's occupational social class as numeric (not centred or transformed) during the survival analyses, rather than ordinal. This was done because use of the ordinal variable violated the proportional hazards assumption, and use of a numeric variable provided similar model fit to a categorical variable.

Patient and public involvement

This study was based on analysis of previously collected and anonymised research data and routinely-collected health data. As such, neither participants nor the public were involved in the design or planning of this study.

Results

Descriptive statistics for the analytic sample, split by father's occupational social class, are shown in Table 1. Mortality status ($p < 0.001$), survival times ($p < 0.001$) and MHT scores ($p < 0.001$) significantly differed between social classes, with a higher proportion of those in higher social classes being alive at censor date (Professional = 69.57%, Unskilled = 55.64%), surviving longer (Professional vs. Unskilled Mean difference = 3.74 years), and demonstrating higher MHT scores (Professional vs. Unskilled Mean difference = 16.97). Note, however, that the mean survival time of individuals with fathers from skilled occupations was lower than those with fathers from semi-skilled occupations, despite a lower proportion of deceased individuals. Furthermore, there were relatively few individuals with a father from a professional occupation. MHT scores were significantly and positively correlated with both father's occupational social class (Spearman's rho = 0.19, $p < 0.001$)

For peer review only - http://bmjopen.bmj.com/site/about/guidelines.xhtml

and survival time (Spearman's rho = 0.07, $p < 0.001$). Likewise, social class was significantly and positively correlated with survival time (Spearman's rho = 0.04, $p < 0.01$).

For peer review only

For peer review only - http://bmjopen.bmj.com/site/about/guidelines.xhtml

Table 1. Descriptive statistics for the analytic sample, split by father's occupational social class.

	Unskilled (N = 1073)	Semi-skilled (N = 1026)	Skilled (N = 2701)	Intermediate (N = 472)	Professional (N = 46)	Total (N = 5318)
**Sex**						
N (%) Male	546 (50.89%)	530 (51.66%)	1332 (49.32%)	239 (50.64%)	21 (45.65%)	2668 (50.17%)
N (%) Female	527 (49.11%)	496 (48.34%)	1369 (50.68%)	233 (49.36%)	25 (54.35%)	2650 (49.83%)
**Moray House Test of Intelligence Score (range = 0-74)** Mean (SD)	31.88 (15.39)	33.67 (15.57)	38.21 (15.18)	39.74 (15.91)	48.85 (12.69)	36.29 (15.65)
**Mortality status**						
N (%) Alive	597 (55.64%)	632 (61.60%)	1703 (63.05%)	321 (68.01%)	32 (69.57%)	3285 (61.77%)
N (%) Dead	476 (44.36%)	394 (38.40%)	998 (36.95%)	151 (31.99%)	14 (30.43%)	2033 (38.23%)
**Time from SMS1947 to death/censor (years)** Mean (SD)	57.14 (16.82)	57.78 (17.01)	56.79 (18.16)	59.96 (15.65)	60.88 (16.62)	57.37 (17.47)

SMS1947 = Scottish Mental Survey 1947

For peer review only - http://bmjopen.bmj.com/site/about/guidelines.xhtml

We then examined selection effects by comparing MHT scores and social class between the analytic sample and those removed due to having missing key variables. Note that this comparison necessarily included only those with at least one complete variable (e.g., a complete MHT score but missing father's social class). Removed individuals had significantly lower MHT score (removed mean = 34.42, $t(454) = -2.22$, $p < 0.05$), but the samples did not significantly differ in terms of social class ($X^2 = 9.30$, $p = 0.05$). This trend has been reported elsewhere (24,25) and may indicate that individuals with lower childhood cognitive ability were more likely to have long-term unemployed fathers who could not be ascribed an occupational social class. Note, however, that differences in MHT score were small; removed individuals scored on average 1.87 points fewer on the MHT.

Returning to the analytic sample, we next compared descriptive statistics between those alive (N = 3285) and those dead (N = 2033) (Table 1). In terms of sex, proportionately more males had died than females ($p < 0.001$). Those who died exhibited significantly lower MHT scores (M = 33.40, SD = 15.89) and tended to be from more manual social classes (Unskilled = 23.41%) than those who survived (MHT: M = 38.07, SD = 15.23, $p < 0.001$; Social class: Unskilled = 18.17%, $p < 0.001$).

We then conducted survival analyses aimed at examining whether social class moderated the association between childhood cognitive ability and all-cause mortality risk. Results from the Schoenfeld test and visual inspection of the scaled Schoenfeld residuals supported the proportional hazard assumption for all predictors in each model (once ordinal social class was replaced with numeric social class). Kaplan-Meier curves showing the association between IQ and survival within each social class are shown in Figure 1.

--Insert Figure 1 here--

For peer review only - http://bmjopen.bmj.com/site/about/guidelines.xhtml

Before examining moderation effects, we tested the independent associations of age-11 IQ and father's social class with all-cause mortality risk, as in previous work. In the univariate models, a 1SD advantage in age-11 IQ predicted a 19% lower mortality risk over time (Table 2). A 1-class advantage in father's social class predicted an 11% lower mortality risk over time. The strength of these associations was similar after adjusting for sex and age at Scottish Mental Survey 1947, though adding sex and age significantly improved the fit of each model (ps < 0.001). Likewise, mutual-adjustment (including sex and age) did not wholly or partly attenuate any one association with mortality risk, and significantly improved model fit versus both sex-adjusted models (ps < 0.001). A 1SD advantage in age-11 IQ and a 1-class advantage in father's social class were both independently associated with small-moderate reductions in all-cause mortality risk (18% and 7%, respectively; Table 2). Note that bias analyses suggested that any unmeasured confounder would require a strong association with the predictors and mortality risk in order to fully account for their association in the present study – particularly the IQ-mortality association (Supplementary Material).

For peer review only - http://bmjopen.bmj.com/site/about/guidelines.xhtml

Table 2. Results from weighted Cox regression models predicting all-cause mortality risk. Regression coefficients and hazard ratios (HR) for IQ score and Father's social class in univariate models, models adjusted for sex and age at Scottish Mental Survey 1947 and models adjusted for both IQ score and Father's social class (and sex and age).

	Univariate[1,2]				Sex and age-adjusted[3,4]				Mutually-adjusted[5]			
**Predictor**	**B**	**SE**	**HR [95% CI]**	**p**	**B**	**SE**	**HR [95% CI]**	**p**	**B**	**SE**	**HR [95% CI]**	**p**
IQ score (1 SD)	-0.21	0.02	0.81 [0.77-0.84]	<0.001	-0.21	0.02	0.81 [0.78-0.85]	<0.001	-0.19	0.02	0.82 [0.79-0.86]	<0.001
Father's social class (1 class)	-0.12	0.02	0.89 [0.85-0.93]	<0.001	-0.12	0.02	0.89 [0.85-0.93]	<0.001	-0.08	0.02	0.93 [0.88-0.97]	<0.001

[1]Univariate IQ model R^2 = 0.02; [2]Univariate Father's social class model R^2 = 0.01; [3]Sex and age-adjusted IQ model R^2 = 0.04; [4]Sex and age-adjusted Father's social class model R^2 = 0.02; [5]Mutually-adjusted model R^2 = 0.04.

For peer review only - http://bmjopen.bmj.com/site/about/guidelines.xhtml

We then tested the moderating effect of father's social class. Adding the interaction term (age-11 IQ by father's social class) did not significantly improve model fit versus the mutually-adjusted model (Mutually-adjusted AIC = 35269.44, Interaction AIC = 35270.08; $X^2\Delta$ = 1.36, p = 0.24; Interaction R^2 = 0.04). The interaction effect did not significantly predict all-cause mortality risk, with 95% CIs that included 1 (B = 0.03, SE = 0.02, HR = 1.03, 95% CI [0.98-1.07], p = 0.25). Adding the interaction term further introduced a multicollinearity issue (VIF = 6.86) – strong collinearity between predictors (sex, father's social class, age-11 IQ and the interaction term) indicating some redundancy – which was not present in the mutually-adjusted model (all VIFs < 1.07).

Finally, we examined the association between age-11 IQ and all-cause mortality risk in each of the social classes (Table 3). A 1SD advantage in age-11 IQ significantly predicted moderately-reduced mortality risk among those from unskilled (22% decrease), semi-skilled (17% decrease) and skilled (17% decrease) backgrounds. The same direction of association, albeit not statistically significant and with wider 95% CIs that included 1, was observed among those from intermediate backgrounds. There was also no significant association between age-11 IQ and mortality risk among those from professional backgrounds, though this group was notably small (N = 46). Confidence intervals for this group were much wider than for other social classes. Although not part of the pre-registered analyses plan, at the advice of a reviewer we examined IQ-mortality associations among those removed individuals with a missing social class (but complete other variables) as these individuals may represent a specific at-risk group. There was a marginally-significant IQ-mortality association such that a 1SD advantage in IQ was associated with a 27% decrease in mortality risk. Note, however, that this group was particularly variable – with wide confidence intervals and a large standard error – reflecting its heterogeneity.

Table 3. Regression coefficients and hazard ratios (HR) for IQ score, adjusted for sex and age at Scottish Mental Survey 1947, in each of the Father's social classes.

	B	SE	HR [95% CI]	p	R^2	AIC
Unskilled (N = 1073)	-0.25	0.05	0.78 [0.71-0.85]	<0.001	0.05	6668.66
Semi-skilled (N = 1026)	-0.19	0.05	0.83 [0.75-0.91]	<0.001	0.03	5526.34

For peer review only - http://bmjopen.bmj.com/site/about/guidelines.xhtml

Skilled (N = 2701)	-0.19	0.03	0.83 [0.78-0.88]	<0.001	0.03	15797.24
Intermediate (N = 472)	-0.13	0.08	0.88 [0.75-1.03]	0.12	0.03	1873.17
Professional (N = 46)	0.13	0.35	1.13 [0.57-2.26]	0.72	0.05	112.63
Missing social class (N = 109)[1]	-0.32	0.17	0.73 [0.52, 1.01]	0.06	0.04	319.87

[1]Includes individuals with missing father's social class but with complete MHT scores and vitality status.

In further exploratory analyses suggested by reviewers we observed no significant interaction between age-11 IQ and father's social class when not adjusting for sex or age at Scottish Mental Survey 1947 as confounders (Supplementary Material). We also observed no significant 3-way interaction between IQ z-score, father's social class and sex (Supplementary Material). Furthermore, a consistent pattern of results was observed when re-analysing the data using additive regression models (Supplementary Material).

Discussion

The present study aimed to establish whether the association between higher childhood cognitive ability and reduced all-cause mortality risk is moderated by childhood socioeconomic circumstances. Whereas advantage in terms of childhood cognitive ability and father's social class independently predicted reduced risk of all-cause mortality, there was no evidence of a significant interaction effect in the whole-sample analysis. Although, in the sub-group analysis, we did observe a significant association between childhood cognitive ability-mortality risk in lower but not higher socioeconomic backgrounds we did not explicitly compare associations between classes. Examination of the absolute difference in the risk of mortality likewise demonstrated that higher childhood cognitive ability and higher childhood socioeconomic circumstances significantly predicted fewer deaths per 10,000 person years, and that the interaction did not significantly predict additional deaths (Supplementary Material). These results support previous observations that advantage in cognitive ability and socioeconomic circumstances have separate contributions to lower all-cause mortality

For peer review only - http://bmjopen.bmj.com/site/about/guidelines.xhtml

risk (6,7), and further suggests that the benefits of high cognitive ability apply regardless of social class.

The lack of a significant moderation effect by socioeconomic circumstances is contrary to our hypothesis and the few previous studies to examine this (12,13). Notably, Jokela et al. (13) observed a significant (albeit small) moderating effect of parental education, with a stronger cognitive ability-mortality association among those with more educated parents, rather than occupational social class as used here. Whereas parental education is thought to reflect the intellectual resources of the family, parental occupation is more indicative of the material resources of the family (26). The benefits of higher cognitive ability for health, therefore, may not depend on the material resources available in childhood. Whereas Kajantie et al. (12) did observe a significant moderation effect using parental occupational social class, only two categories were used (manual and middle-class). Such a coarse measure of occupational social class may overestimate its contribution to all-cause mortality risk by shifting the estimates for each category towards the mean of included classes – downwards for lower social classes and upwards for higher social classes. Furthermore, it assumes no differences between social classes within each category; here, the mortality risk associated with skilled, intermediate and professional occupational classes was very different.

The main strength of the present study is the use of both socioeconomic circumstances and cognitive ability measured contemporaneously in childhood. In the present study, advantage in these factors independently predicted reduced mortality risk, with no evidence of multicollinearity or confounding by sex. Although we only included sex as a potential confounder, a more thorough investigation of bias using other potential confounders and mediators is available elsewhere (9–11), including in the same Scottish Mental Survey 1947 subsamples (6,7,14,15). Notably, childhood cognitive ability and childhood socioeconomic circumstances have been shown to predict later-life health, even after accounting for mediators such as education and socioeconomic circumstances in adulthood (5,6,25,27). Measuring these factors contemporaneously avoids potential recall bias

For peer review only - http://bmjopen.bmj.com/site/about/guidelines.xhtml

associated with retrospective measures of socioeconomic circumstances in particular (28). Also, as individuals must survive until cognitive and socioeconomic measures are taken, examining these factors in childhood lessens potential selection effects (e.g., lower cognitive ability individuals who may die prior to adulthood). Furthermore, the present study examined all-cause mortality risk in a large sample over an entire life course (age 11-79 years-old). These individuals have had longer exposure to mortality risk than the younger samples used in previous work (12,13).

A limitation is that the sample is a product of the time and place in which they lived (29), and this may limit the generalisability of the results. All individuals in the sample were born in 1936 and lived in Scotland in 1947. They survived the Second World War and lived through the significant social, economic and educational changes that followed. Note that this also applies to previous work using the Helsinki Birth Cohort (born 1934-1944)(12). Childhood socioeconomic conditions (including occupational and educational opportunities of parents) in more modern samples, such as in the US National Longitudinal Survey of Youth(13), are likely better overall than those captured in the present sample (30). Indeed, relatively few individuals here originated from higher childhood social classes (intermediate or professional occupations). Furthermore, removal of individuals with missing childhood cognitive ability, childhood social class, or vitality status likely resulted in a sample slightly biased towards higher cognitive ability. Note also that the sample selection analyses does not include those with no complete key variables (i.e., all missing), who may constitute a very different population.

There are further limitations arising due to our use of father's occupational social class as a proxy measure of socioeconomic conditions. Firstly, although deceased, absent and long-term unemployed fathers were coded according to their most recent occupation where possible, the removal of individuals with a missing father's occupational social class likely results in a sample that does not represent the full range of socioeconomic conditions, though this is not a limitation that is unique to this study (12). Individuals raised in single parent households have been shown to be at higher risk of

For peer review only - http://bmjopen.bmj.com/site/about/guidelines.xhtml

mortality (31), so removed individuals may represent a particularly vulnerable population. Secondly, while father's occupational social class is useful for this age of cohort it is perhaps less useful for younger cohorts for whom fathers are less likely to be the main breadwinners. More generally, the meaning of occupational social class is likely to have changed as new occupations are created and working conditions improved (32), thus the nature of the role of social class may be different in more modern social classes. It is unclear whether other measures of childhood socioeconomic circumstances, such as parental education, play a role in moderating the benefits of higher childhood cognitive ability. Furthermore, these individuals had access to universal healthcare throughout their lives, unlike previous work using US-based samples (13), likely reducing the importance of socioeconomic circumstances as both a predictor of mortality (33) and a moderator of the cognitive ability-mortality association. Finally, there are a number of unmeasured confounders, such as childhood health and earlier socioeconomic conditions, which may impact both childhood cognitive ability and mortality risk (9). For example, socioeconomic disadvantage around birth (e.g., lower family income and parental education) may lead to both poorer childhood cognitive ability (34) and increased mortality risk (35), resulting in an arbitrary but strong association between higher cognitive ability and reduced mortality risk. However, previous work has shown that accounting for potential confounders such as earlier socioeconomic circumstances (including father's social class and quality of home environment) and childhood health results in only a small attenuation of the association between higher childhood cognitive ability and reduced mortality risk (9). Furthermore, sensitivity analysis suggested that a particularly strong unmeasured confounder would be required to wholly account for the IQ-mortality association observed in the present study (Supplementary Material).

Consistent with previous work, the present study demonstrates an association between higher cognitive ability and lower all-cause mortality risk. However, it also suggests that this association is not conditional on one's social class of origin. Whereas childhood social class was an important independent predictor of all-cause mortality risk, there was no evidence that it moderated the role

For peer review only - http://bmjopen.bmj.com/site/about/guidelines.xhtml

of childhood cognitive ability in predicting longevity. The benefits of higher childhood cognitive ability for lower all-cause mortality risk were observed across childhood social classes. This is an important consideration for public health policy and interventions: childhood cognitive ability is a source of health inequality independent from material socioeconomic resources in childhood, and needs to be addressed separately. Future work should focus on building causal evidence and clarifying the mechanisms by which childhood cognitive ability and childhood socioeconomic conditions affect mortality risk.

Contributors

All authors contributed to the conceptualization and design of this study. MHI contributed to data curation and formal analysis. MHI, DA and IJD contributed to investigation, methodology, project administration, validation, visualisation, writing – original draft preparation, and writing – review & editing. IJD contributed to funding acquisition and supervision.

Data sharing

Historical data from the Scottish Mental Survey 1947 is available upon request from the Department of Psychology, The University of Edinburgh. Access is contingent on local governance approvals. Routinely-collected records, including national death records, are available and linkable through NHS Information Services Division and the eData Research and Innovation Service (https://www.isdscotland.org/Products-and-Services/EDRIS/). Access is contingent on ethical and Public Benefit and Privacy Panel approvals.

Ethics statement

For peer review only - http://bmjopen.bmj.com/site/about/guidelines.xhtml

This submission is covered by the ethics application "Lifelong health and wellbeing of the 'Scotland in Miniature' cohort: The 6-Day Sample of the Scottish Mental Survey" (REC# 12/SS/0024; Scotland A Research Ethics Committee).

Competing interests

The authors declare no competing interests, no financial relationships with any organisations that might have an interest in the submitted work, and no other relationships or activities that could appear to have influenced the submitted work.

Funding statement

This work was supported by a Medical Research Council Mental Health Data Pathfinder award [MRC-MC_PC_17209], and by a UK cross council Lifelong Health and Wellbeing Initiative [MRCG1001401], for which IJD is the principal investigator. MHI, DA and IJD are members of The University of Edinburgh Centre for Cognitive Ageing and Cognitive Epidemiology, part of the cross council Lifelong Health and Wellbeing Initiative [MR/K026992/1].

For peer review only - http://bmjopen.bmj.com/site/about/guidelines.xhtml

References

1. Korten AE, Jorm AF, Jiao Z, Letenneur L, Jacomb PA, Henderson AS, et al. Health, cognitive, and psychosocial factors as predictors of mortality in an elderly community sample. J Epidemiol Community Health. 1999;53(2):83–8.

2. Pavlik VN, De Moraes SA, Szklo M, Knopman DS, Mosley TH, Hymans DJ. Relation between cognitive function and mortality in middle-aged adults: The atherosclerosis risk in communities study. Am J Epidemiol. 2003;157(4):327–34.

3. Batterham PJ, Christensen H, Mackinnon AJ. Fluid intelligence is independently associated with all-cause mortality over 17 years in an elderly community sample: An investigation of potential mechanisms. Intelligence [Internet]. 2009;37(6):551–60. Available from: http://dx.doi.org/10.1016/j.intell.2008.10.004

4. Bratsberg B, Rogeberg O. Childhood socioeconomic status does not explain the IQ-mortality gradient. Intelligence [Internet]. 2017;62(March):148–54. Available from: http://dx.doi.org/10.1016/j.intell.2017.04.002

5. Calvin CM, Deary IJ, Fenton C, Roberts BA, Der G, Leckenby N, et al. Intelligence in youth and all-cause-mortality: Systematic review with meta-analysis. Int J Epidemiol. 2011;40(3):626–44.

6. Calvin CM, Batty GD, Der G, Brett CE, Taylor A, Pattie A, et al. Childhood intelligence in relation to major causes of death in 68 year follow-up: Prospective population study. BMJ. 2017;357.

7. Iveson MH, Čukič I, Der G, Batty GD, Deary IJ. Intelligence and all-cause mortality in the 6-Day Sample of the Scottish Mental Survey 1947 and their siblings: Testing the contribution of family background. Int J Epidemiol. 2018;47(1).

8. Whalley LJ, Deary IJ. Longitudinal cohort study of childhood IQ and survival up to age 76. Br

For peer review only - http://bmjopen.bmj.com/site/about/guidelines.xhtml

Med J. 2001;322(7290):819–22.

9. Kuh D, Richards M, Hardy R, Butterworth S, Wadsworth MEJ. Childhood cognitive ability and deaths up until middle age: A post-war birth cohort study. Int J Epidemiol. 2004;33(2):408–13.

10. Batty GD, Wennerstad KM, Smith GD, Gunnell D, Deary IJ, Tynelius P, et al. IQ in early adulthood and mortality by middle age: Cohort study of 1 million swedish men. Epidemiology. 2009;20(1):100–9.

11. Lager A, Bremberg S, Vågerö D. The association of early IQ and education with mortality: 65 Year longitudinal study in Malmö, Sweden. BMJ. 2009;339(7735):1432.

12. Kajantie E, Räikkönen K, Henriksson M, Forsén T, Heinonen K, Pesonen AK, et al. Childhood socioeconomic status modifies the association between intellectual abilities at age 20 and mortality in later life. J Epidemiol Community Health. 2010;64(11):963–9.

13. Jokela M, Elovainio M, Singh-Manoux A, Kivimäki M. IQ, Socioeconomic status, and early death: The US national longitudinal survey of youth. Psychosom Med. 2009;71(3):322–8.

14. Deary IJ, Brett CE. Predicting and retrodicting intelligence between childhood and old age in the 6-Day Sample of the Scottish Mental Survey 1947. Intelligence [Internet]. 2015;50:1–9. Available from: http://dx.doi.org/10.1016/j.intell.2015.02.002

15. Čukić I, Brett CE, Calvin CM, Batty GD, Deary IJ. Childhood IQ and survival to 79: Follow-up of 94% of the Scottish Mental Survey 1947. Intelligence. 2017;63(May):45–50.

16. The Scottish Council for Research in Education. The trend of Scottish intelligence. London: University of London Press; 1949.

17. Brett CE, Deary IJ. Realising health data linkage from a researcher's perspective: following up the 6-Day Sample of the Scottish Mental Survey 1947. Longit Life Course Stud. 2014;5(3):283–

For peer review only - http://bmjopen.bmj.com/site/about/guidelines.xhtml

98.

18. Deary IJ, Whiteman MC, Starr JM, Whalley LJ, Fox HC. The Impact of Childhood Intelligence on Later Life: Following Up the Scottish Mental Surveys of 1932 and 1947. J Pers Soc Psychol. 2004;86(1):130–47.

19. General Register Office. Census, 1951: Classification of Occupations. London: HMSO; 1956.

20. R Core Team. R: A language and environment for statistical computing. Vienna: R Foundation for Statistical Computing; 2018.

21. RStudio Team. RStudio: Integrated development for R. Boston, MA: RStudio Inc.; 2016.

22. Revelle W. psych: Procedures for personality and psychological research. Evanston: Northwestern University; 2018.

23. Therneau TM, Lumley T. Package "survival." R Top Doc. 2015;128.

24. Johnson W, Brett CE, Calvin C, Deary IJ. Childhood characteristics and participation in Scottish Mental Survey 1947 6-Day Sample Follow-ups: Implications for participation in aging studies. Intelligence [Internet]. 2016;54:70–9. Available from: http://dx.doi.org/10.1016/j.intell.2015.11.006

25. Iveson MH, Deary IJ. Intergenerational social mobility and subjective wellbeing in later life. Soc Sci Med [Internet]. 2017;188:11–20. Available from: http://dx.doi.org/10.1016/j.socscimed.2017.06.038

26. Galobardes B, Shaw M, Lawlor DA, Lynch JW, Smith GD. Indicators of socioeconomic position (part 1). J Epidemiol Community Health. 2006;60(1):7–12.

27. Iveson MH, Deary IJ. Early-life predictors of retirement decisions and post-retirement health. SSM - Popul Heal [Internet]. 2019;8(February):100430. Available from: https://doi.org/10.1016/j.ssmph.2019.100430

For peer review only

For peer review only - http://bmjopen.bmj.com/site/about/guidelines.xhtml

28. Batty GD, Lawlor DA, Macintyre S, Clark H, Leon DA. Accuracy of adults' recall of childhood social class: Findings from the Aberdeen children of the 1950s study. J Epidemiol Community Health. 2005;59(10):898–903.

29. Elder Jr GH. Time, human agency, and social change: Perspectives on the life course. Soc Psychol Q. 1994;4–15.

30. Galobardes B, Lynch JW, Smith GD. Childhood socioeconomic circumstances and cause-specific mortality in adulthood: Systematic review and interpretation. Epidemiol Rev. 2004;26:7–21.

31. Weitoft GR, Hjern A, Haglund B, Rosén M. Mortality, severe morbidity, and injury in children living with single parents in Sweden: A population-based study. Lancet. 2003;361(9354):289–95.

32. Goldthorpe JH. On Economic Development and Social Mobility. Br J Soc. 1985;36(4):549–73.

33. Asaria M, Ali S, Doran T, Ferguson B, Fleetcroft R, Goddard M, et al. How a universal health system reduces inequalities: Lessons from England. J Epidemiol Community Health. 2016;70(7):637–43.

34. Lawlor DA, Batty GD, Morton SMB, Deary IJ, Macintyre S, Ronalds G, et al. Early life predictors of childhood intelligence: Evidence from the Aberdeen children of the 1950s study. J Epidemiol Community Health. 2005;59(8):656–63.

35. Osler M, Andersen a-M, Due P, Lund R, Damsgaard M, Holstein B. longitudinal study of Danish men born in 1953. J Epidemiol Community Heal. 2003;57:681–6.

For peer review only

Figure 1. Kaplan-Meier Survival curves showing survival probability associated with IQ z-scores 1 SD below (red), around (green) or above (blue) the mean, split by father's occupational social class.

For peer review only

For peer review only - http://bmjopen.bmj.com/site/about/guidelines.xhtml

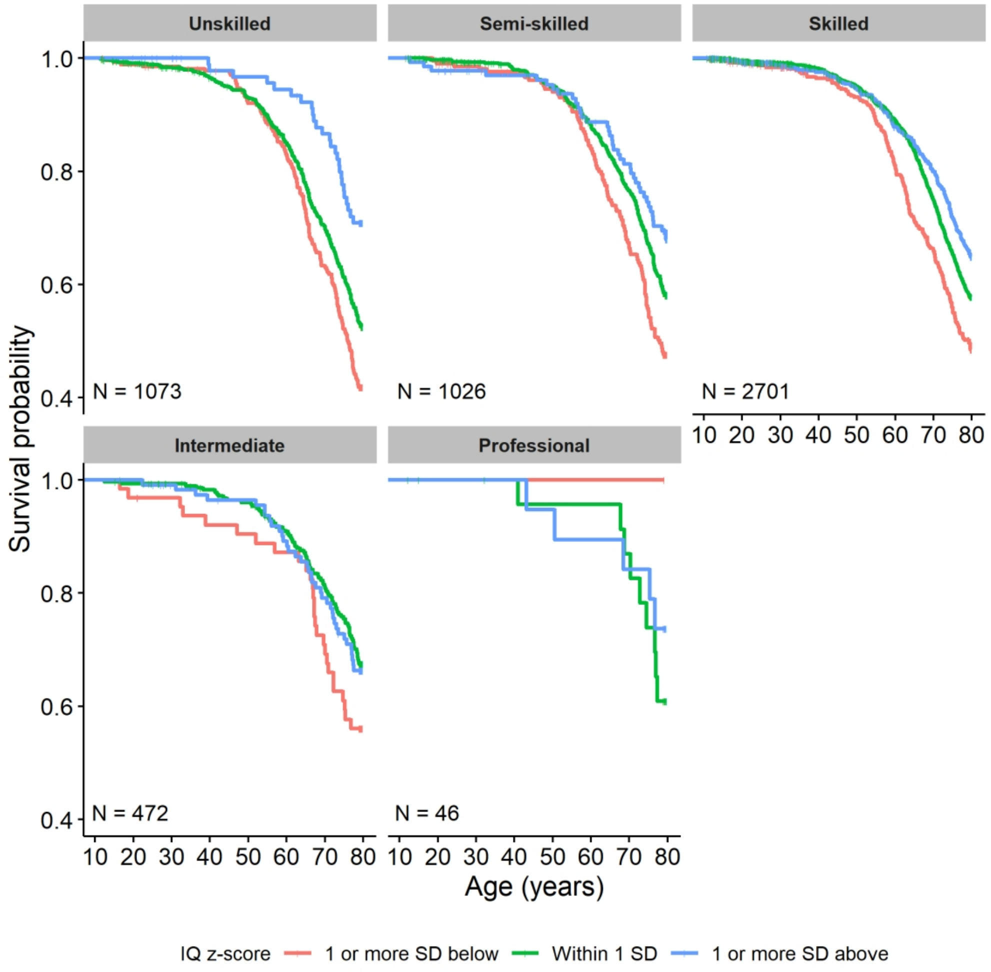

Figure 1. Kaplan-Meier Survival curves showing survival probability associated with IQ z-scores 1 SD below (red), around (green) or above (blue) the mean, split by father's occupational social class.

179x179mm (300 x 300 DPI)

Supplementary Material

Bias analyses

Although the analyses presented in the main text incorporates sex and age as covariates, there is the potential for unmeasured factors to confound the observed associations with mortality risk. To account for this, we calculated E-values to estimate the minimum strength of association that an unmeasured confounder would need to have with each predictor (age-11 IQ or father's social class) and mortality risk, on the hazard ratio (HR) scale, to fully explain the observed associations. Large E-values indicate that strong confounding would be required to fully account for a given association. Notably, it is recommended that E-values are reported both for a given point estimate HR and for its 95% CI boundary closest to the null (1 on the HR scale)(1).

Based on the univariate association between age-11 IQ and mortality risk (Main Text, Table 2), we estimated an E-value of 1.58 (E-value for upper limit of 95% CI = 1.51). Based on the sex- and age-adjusted association between age-11 IQ and mortality risk (Main Text, Table 2), we estimated an E-value of 1.58 (E-value for upper limit of 95% CI = 1.48). Finally, based on the point estimate of the mutually-adjusted (sex, age and father's social class) association between age-11 IQ and mortality risk (Main Text, Table 2), we estimated an E-value of 1.56 (E-value for upper limit of 95% CI = 1.46).

Based on the univariate association between father's social class and mortality risk (Main Text, Table 2), we estimated an E-value of 1.39 (E-value for upper limit of 95% CI = 1.28). Based on the sex- and age-adjusted association between father's social class and mortality risk (Main Text, Table 2), we estimated an E-value of 1.39 (E-value for upper limit of 95% CI = 1.28). Finally, based on the point estimate of the mutually-adjusted (sex, age and father's social class) association between father's social class and mortality risk (Main Text, Table 2), we estimated an E-value of 1.28 (E-value for upper limit of 95% CI = 1.17).

In the context of the associations observed in the present study, this analysis suggests that a potential confounder would need to have a relatively strong association with the predictors and mortality risk to fully explain their association, particularly the association between age-11 IQ and mortality risk.

Unadjusted interaction analyses

The interaction analyses reported in the main text are adjusted for sex and age in days at Scottish Mental Survey 1947. In the analyses presented below (Table A1) we re-estimate the mutually-adjusted model (including IQ score and father's social class) and the interaction model (including the IQ X social class interaction) without adjusting for these potential confounders, and with adjusting for only one confounder (sex or age).

For peer review only - http://bmjopen.bmj.com/site/about/guidelines.xhtml

Adding the IQ-social class interaction term did not significantly improve model fit versus the corresponding main effects model (Unadjusted: AIC = 35357.72, $X^2\Delta = 0.95$, p = 0.33; Sex-adjusted: AIC = 35270.27, $X^2\Delta = 1.36$, p = 0.24; Age-adjusted: AIC = 35356.28, $X^2\Delta = 0.96$, p = 0.33). Furthermore, the interaction did not significantly predict all-cause mortality in any of the models (see Table A1).

For peer review only

Table A1. Results from weighted Cox regression models predicting all-cause mortality risk. Regression coefficients and hazard ratios (HR) for IQ score and Father's social class in main effects only model and the interaction model without further adjustment for covariates, adjusted for sex only, and adjusted for age at Scottish Mental Survey 1947 only.

	Unadjusted[1,2]				Sex-adjusted[3,4]				Age-adjusted[5,6]			
**Predictor**	**B**	**SE**	**HR [95% CI]**	**p**	**B**	**SE**	**HR [95% CI]**	**p**	**B**	**SE**	**HR [95% CI]**	**p**
Main effects only model												
IQ score (1 SD)	-0.20	0.02	0.82 [0.79-0.86]	<0.001	-0.19	0.02	0.83 [0.79-0.86]	<0.001	-0.21	0.02	0.81 [0.78-0.85]	<0.001
Father's social class (1 class)	-0.08	0.02	0.93 [0.89-0.97]	<0.001	-0.08	0.02	0.92 [0.88-0.97]	<0.001	-0.07	0.02	0.93 [0.89-0.97]	<0.01
Interaction model												
IQ score X Father's social class	0.02	0.02	1.02 [0.98-1.07]	0.33	0.03	0.02	1.03 [0.98-1.07]	0.24	0.02	0.02	1.02 [0.98-1.07]	0.33

[1]Unadjusted main effects only model R^2 = 0.02; [2]Unadjusted interaction model R^2 = 0.02; [3]Sex-adjusted main effects only mode R^2 = 0.04; [4]Sex-adjusted interaction model R^2 = 0.04; [5]Age-adjusted main effects only model R^2 = 0.02, [6]Age-adjusted interaction model R^2 = 0.02.

For peer review only - http://bmjopen.bmj.com/site/about/guidelines.xhtml

3-way Interaction

At the request of a reviewer, we additionally performed an exploratory analysis of a three-way interaction between IQ z-scores, father's occupational social class (reversed, continuous) and sex. In particular, the aim was to test whether the moderating role of childhood socioeconomic circumstances itself depended on sex.

This was done by constructing a Cox regression model that included main effects of IQ z-scores, father's social class (reversed, continuous), sex and age in days at the Scottish Mental Survey 1947, two-way interactions between IQ z-scores X father's social class, IQ z-scores X sex and father's social class X sex, as well as the three-way interaction between IQ z-scores X father's social class X sex.

Including these additional interaction effects resulted in multicollinearity issues (all interaction VIFs > 8.50). Furthermore, it did not significantly improve model fit versus the IQ z-score X father's social class model included in the main text (AIC = 35275.82; $X^2\Delta = 0.26$, p = 0.97; Interaction $R^2 = 0.04$). None of the included interactions significantly predicted all-cause mortality risk (Table A2).

Table A2. Results from weighted Cox regression model, adjusted for age in days at Scottish Mental Survey 1947, predicting all-cause mortality risk. Regression coefficients and hazard ratios (HR) for IQ score, Father's social class, Sex, IQ X Father's social class, IQ X Sex, Father's social class X Sex, IQ X Father's social class X Sex.

**Predictor**	**B**	**SE**	**HR [95% CI]**	**p**
IQ score (1 SD)	-0.26	0.07	0.77 [0.67-0.89]	<0.001
Father's social class (1 class)	-0.07	0.03	0.94 [0.88-1.00]	0.04
Sex (Female)	-0.36	0.13	0.70 [0.54-0.90]	<0.01
IQ X Father's social class	0.02	0.03	1.03 [0.97-1.08]	0.38
IQ X Sex	0.01	0.12	1.01 [0.80-1.27]	0.94
Father's social class X Sex	-0.02	0.05	0.98 [0.89-1.08]	0.70
IQ X Father's social class X Sex	<0.01	0.04	1.00 [0.92-1.10]	0.94

Additive hazards model

At the request of a reviewer, we performed Additive Hazards regression using Aalen's additive regression models equivalent to the Cox Proportional Hazards models used in the main text. Additive models have the advantage of allowing the effects of covariates – including interaction effects – to vary over time (2). For example, advantage in childhood cognitive ability may benefit survival in particular socioeconomic groups but only in specific periods of life. Additive models help to provide information about the effect in the context of the underlying hazard – a small hazard ratio for the interaction may still be important if the

For peer review only - http://bmjopen.bmj.com/site/about/guidelines.xhtml

underlying hazard is large (2). We estimated the number of additional deaths per 10,000 person years predicted by each covariate of interest – IQ z-scores, father's occupational social class and the IQ-social class interaction. As in the main text, univariate, sex and age-adjusted, mutually-adjusted, and interaction models were constructed.

As with mortality risk in the main text, a 1SD advantage in IQ and a 1 class advantage in father's social class were both significantly associated with a small reduction in the number of deaths, even when adjusted for sex and age at time of the Scottish Mental Survey 1947 (Table A3). These associations remained significant when mutually-adjusting, supporting the suggestion that childhood cognitive ability and childhood socioeconomic circumstances independently predict mortality risk.

We then tested the moderating effect of father's social class. The IQ-social class interaction predicted a small increase of 0.13 deaths per 10,000 person years, though this was only marginally-significant and 95% CIs included 0 (95% CI [0.00-0.25], $p = 0.05$). That is, a 1SD advantage in childhood cognitive ability predicted 0.13 more deaths per increase in father's social class. Note that this association was much smaller in magnitude than the decreases in additional deaths associated with the main effects.

Additive models were constructed to examine the IQ-mortality association within each social class, including those whose father's occupational social class was missing, as in the main text (Table A4). The direction and pattern of associations was consistent with the Cox Proportional Hazards models used in the main text: a 1SD advantage in IQ was significantly associated with fewer deaths per person year among those from unskilled, semi-skilled and skilled social class backgrounds. The benefit of advantage in IQ appeared to diminish slightly as class increased, suggesting that high IQ may benefit those from lower social classes most. Note again, however, that the interaction effect in the whole-sample model above was not significant. There was no significant association between IQ and mortality in those from intermediate, professional or missing occupational social classes, and confidence intervals in these groups were wide.

We conclude that the additive hazards approach presented here is in keeping with the proportional hazards approach used in the main text. In the context of the underlying mortality hazard for this sample, advantage in IQ and childhood social class predict modest reductions in the number of deaths per 10,000 person years. In contrast, the interaction effect was relatively weak, with no consistent trend across classes.

For peer review only - http://bmjopen.bmj.com/site/about/guidelines.xhtml

Table A3. Results from weighted Additive Hazards regression models predicting all-cause mortality risk. Additional deaths per 10,000 person years and 95% Confidence Intervals for IQ score and Father's social class in univariate models, models adjusted for sex and age at Scottish Mental Survey 1947, models adjusted for both IQ score and Father's social class (and sex and age), and a model additionally including the IQ-Social class interaction effect (and age and sex).

	Univariate		Sex and age-adjusted		Mutually-adjusted	
**Predictor**	**Additional deaths per 10,000 person years [95% CI]**	**p**	**Additional deaths per 10,000 person years [95% CI]**	**p**	**Additional deaths per 10,000 person years [95% CI]**	**p**
IQ score (1 SD)	-0.57 [-0.69, -0.45]	<0.001	-0.56 [-0.68, -0.44]	<0.001	-0.53 [-0.66, -0.41]	<0.001
Father's social class (1 class)	-0.31 [-0.43, -0.19]	<0.001	-0.31 [-0.43, -0.19]	<0.001	-0.20 [-0.33, -0.08]	<0.01

Table A4. Additional deaths per 10,000 person years and 95% Confidence Intervals for IQ score, adjusted for sex and age at Scottish Mental Survey 1947, in each of the Father's social classes.

	**Additional deaths per 10,000 person years [95% CI]**	**p**
Unskilled (N = 1073)	-3.37 [-4.58, -2.15]	<0.001
Semi-skilled (N = 1026)	-2.67 [-4.09, -1.24]	<0.001
Skilled (N = 2701)	-1.05 [-1.41, -0.68]	<0.001
Intermediate (N = 472)	-3.62 [-8.26, 1.02]	0.13
Professional (N = 46)	31.16 [-146.18, 208.50]	0.73
Missing social class (N = 109)[1]	-42.56 [-88.32, 3.19]	0.07

[1]Includes individuals with missing father's social class but with complete MHT scores and vitality status.

For peer review only - http://bmjopen.bmj.com/site/about/guidelines.xhtml

References

1. VanderWeele TJ, Ding P. Sensitivity analysis in observational research: Introducing the E-value. Annals of Internal Medicine. 2017;167:268-274. doi: 10.7326/M16-2607
2. Rod NJ, Lange T, Andersen I, Marott JL, Diderichsen F. Additive interaction in survival analysis: Use of the additive hazards model. Epidemiology. 2012;23(5):733-737.

For peer review only

For peer review only - http://bmjopen.bmj.com/site/about/guidelines.xhtml

STROBE Statement—Checklist of items that should be included in reports of ***cohort studies***

	Item No	Recommendation	Page (Line)
**Title and abstract**	1	(*a*) Indicate the study's design with a commonly used term in the title or the abstract	1 (1)
		(*b*) Provide in the abstract an informative and balanced summary of what was done and what was found	2 (1)
**Introduction**			
Background/rationale	2	Explain the scientific background and rationale for the investigation being reported	4 (1)
Objectives	3	State specific objectives, including any prespecified hypotheses	5 (19)
**Methods**			
Study design	4	Present key elements of study design early in the paper	6 (7)
Setting	5	Describe the setting, locations, and relevant dates, including periods of recruitment, exposure, follow-up, and data collection	6 (7)
Participants	6	(*a*) Give the eligibility criteria, and the sources and methods of selection of participants. Describe methods of follow-up	6 (7)
		(*b*) For matched studies, give matching criteria and number of exposed and unexposed	NA
Variables	7	Clearly define all outcomes, exposures, predictors, potential confounders, and effect modifiers. Give diagnostic criteria, if applicable	6 (23)
Data sources/ measurement	8*	For each variable of interest, give sources of data and details of methods of assessment (measurement). Describe comparability of assessment methods if there is more than one group	6 (23)
Bias	9	Describe any efforts to address potential sources of bias	8 (7)
Study size	10	Explain how the study size was arrived at	6 (15)
Quantitative variables	11	Explain how quantitative variables were handled in the analyses. If applicable, describe which groupings were chosen and why	8 (7)
Statistical methods	12	(*a*) Describe all statistical methods, including those used to control for confounding	8 (4)
		(*b*) Describe any methods used to examine subgroups and interactions	8 (17)
		(*c*) Explain how missing data were addressed	6 (15)
		(*d*) If applicable, explain how loss to follow-up was addressed	7 (21)
		(*e*) Describe any sensitivity analyses	NA
**Results**			
Participants	13*	(a) Report numbers of individuals at each stage of study—eg numbers potentially eligible, examined for eligibility, confirmed eligible, included in the study, completing follow-up, and analysed	6 (7)
		(b) Give reasons for non-participation at each stage	6 (15)
		(c) Consider use of a flow diagram	NA
Descriptive data	14*	(a) Give characteristics of study participants (eg demographic, clinical, social) and information on exposures and potential confounders	9 (13)
		(b) Indicate number of participants with missing data for each variable of interest	6 (15)
		(c) Summarise follow-up time (eg, average and total amount)	7 (24)
Outcome data	15*	Report numbers of outcome events or summary measures over time	10
Main results	16	(*a*) Give unadjusted estimates and, if applicable, confounder-adjusted estimates and their precision (eg, 95% confidence interval). Make clear	13

For peer review only

For peer review only - http://bmjopen.bmj.com/site/about/guidelines.xhtml

		which confounders were adjusted for and why they were included	
		(*b*) Report category boundaries when continuous variables were categorized	NA
		(*c*) If relevant, consider translating estimates of relative risk into absolute risk for a meaningful time period	NA
Other analyses	17	Report other analyses done—eg analyses of subgroups and interactions, and sensitivity analyses	14 (1)
**Discussion**			
Key results	18	Summarise key results with reference to study objectives	15 (10)
Limitations	19	Discuss limitations of the study, taking into account sources of potential bias or imprecision. Discuss both direction and magnitude of any potential bias	17 (1)
Interpretation	20	Give a cautious overall interpretation of results considering objectives, limitations, multiplicity of analyses, results from similar studies, and other relevant evidence	18 (5)
Generalisability	21	Discuss the generalisability (external validity) of the study results	17 (1)
**Other information**			
Funding	22	Give the source of funding and the role of the funders for the present study and, if applicable, for the original study on which the present article is based	19 (13)

*Give information separately for exposed and unexposed groups.

Note: An Explanation and Elaboration article discusses each checklist item and gives methodological background and published examples of transparent reporting. The STROBE checklist is best used in conjunction with this article (freely available on the Web sites of PLoS Medicine at http://www.plosmedicine.org/, Annals of Internal Medicine at http://www.annals.org/, and Epidemiology at http://www.epidem.com/). Information on the STROBE Initiative is available at http://www.strobe-statement.org.

For peer review only
